# Nutritional interventions in children with acute lymphoblastic leukemia undergoing antineoplastic treatment: a systematic review

**DOI:** 10.1186/s40795-024-00892-4

**Published:** 2024-06-19

**Authors:** Alan E. Guzmán-León, Jessica Avila-Prado, Leslie R. Bracamontes-Picos, Michelle M. Haby, Katja Stein, Humberto Astiazaran-Garcia, Veronica Lopez-Teros

**Affiliations:** 1 Department of Chemical and Biological Sciences, Universidad de Sonora, Blvd. Luis Encinas y Rosales S/N, Hermosillo , 83000 Sonora, Mexico; 2 School of Population and Global Health, Centre for Health Policy, The University of Melbourne, Melbourne, VIC Australia; 3 Hospital Civil de Guadalajara “Dr. Juan I. Menchaca“, Centro Universitario de Ciencias de La Salud, Universidad de Guadalajara, Guadalajara, Jalisco Mexico; 4 Research Center for Food and Development (CIAD), Sonora, Mexico

**Keywords:** Pediatric acute lymphoblastic leukemia, Nutrition intervention, Malnutrition, Systematic review

## Abstract

**Background:**

A compromised nutritional status jeopardizes a positive prognosis in acute lymphoblastic leukemia (ALL) patients. In low- and middle-income countries, ~ 50% of children with ALL are malnourished at diagnosis time, and undergoing antineoplastic treatment increases the risk of depleting their nutrient stores. Nutrition interventions are implemented in patients with cancer related malnutrition. We aimed to evaluate the effect of nutrition interventions in children diagnosed with ALL under treatment.

**Methods:**

Using a predefined protocol, we searched for published or unpublished randomized controlled trials in: Cochrane CENTRAL, MEDLINE, EMBASE, LILACS, and SciELO, and conducted complementary searches. Studies where at least 50% of participants had an ALL diagnosis in children ≤ 18 years, active antineoplastic treatment, and a nutrition intervention were included. Study selection and data extraction were conducted independently by three reviewers, and assessment of the risk of bias by two reviewers. Results were synthesized in both tabular format and narratively.

**Results:**

Twenty-five studies (out of 4097 records) satisfied the inclusion requirements. There was a high risk of bias in eighteen studies. Interventions analyzed were classified by compound/food (*n* = 14), micronutrient (*n* = 8), and nutritional support (*n* = 3). Within each group the interventions and components (dose and time) tested were heterogeneous. In relation to our primary outcomes, none of the studies reported fat-free mass as an outcome. Inflammatory and metabolic markers related to nutritional status and anthropometric measurements were reported in many studies but varied greatly across the studies. For our secondary outcomes, fat mass or total body water were not reported as an outcome in any of the studies. However, some different adverse events were reported in some studies.

**Conclusions:**

This review highlights the need to conduct high-quality randomized controlled trials for nutrition interventions in children with ALL, based on their limited number and heterogeneous outcomes.

**Registration of the review protocol:**

Guzmán-León AE, Lopez-Teros V, Avila-Prado J, Bracamontes-Picos L, Haby MM, Stein K. Protocol for a Systematic Review: Nutritional interventions in children with acute lymphoblastic leukemia undergoing an tineoplastic treatment. International prospective register of systematic reviews. 2021; PROSPERO CRD:42,021,266,761 (https://www.crd.york.ac.uk/prospero/display_record.php?RecordID=266761).

**Supplementary Information:**

The online version contains supplementary material available at 10.1186/s40795-024-00892-4.

## Background

Leukemia is the most common cancer in children, accounting for 1 ~ 3 cases of pediatric cancer) [1–3]. Additionally, three out of four cases of leukemia in pediatric age correspond to acute lymphoblastic leukemia (ALL) [1–3].

Acute lymphoblastic leukemia (ALL) is a type of cancer that starts in the bone marrow, generating mutations in B or T lymphoid progenitor cells, affecting the ability to proliferate, survive, mature, and accumulate [3, 4]. Treatment for ALL may impact body composition and the patient’s nutritional status [e.g., fat-free mass (FFM) loss, fat mass (FM) increase, fluid retention] [5–10]. According to recent research, children with ALL who have a compromised nutritional condition may be at greater risk of a poor prognosis [6, 11–15]. In low and middle-income countries, about 50% of children with ALL are malnourished at diagnosis [5, 8, 16–19]. The World Health Organization defines malnutrition as the deficiency, excess, or imbalance in energy and/or nutrient intake (e.g., underweight, obesity, and/or micronutrient deficiencies) [20]. Malnutrition manifests as weight changes in its basic form, but the most relevant clinical alterations are seen in body composition (distribution and proportion of FM and FFM) [5, 14, 15, 20, 21].

A higher risk of infections, poor treatment efficacy (e.g., tolerance and adherence), a lower survival rate, and death have also been linked to malnutrition at the time of diagnosis or during antineoplastic treatment (also known as anticancer, chemotherapy, chemo, cytotoxic, or hazardous drugs) [5, 17, 22–24]. This could be associated with malnutrition mediated changes, such as inflammation, increased energy expenditure, low or excessive caloric intake, and alterations in metabolic pathways [5, 14, 21, 25].

Due to medication side effects, pediatric patients with ALL undergoing antineoplastic therapy may experience decreased oral food intake/tolerance or increased losses (e.g., vomiting, diarrhea, or renal losses), increasing the risk of malnutrition [19, 21, 26]. Additionally, the use of steroids during treatment might be accompanied by increased intake of energy-dense foods with low nutritional value, increasing the risk of weight gain and micronutrient deficiencies [5, 12, 27, 28].

Meeting nutritional requirements in pediatric patients with ALL is challenging, frequently leading to the use of oral, enteral, or parenteral nutritional support, to compensate for oral intolerance [14, 19, 26, 29–31]. These nutrition interventions aim to prevent/control/reverse malnutrition complications associated with the antineoplastic treatment, while promoting normal growth [11, 24, 32], improving the quality of life, treatment tolerance, and immunocompetence [26, 33].

To date, only two published systematic reviews focus on nutritional support in cancer patients (including patients with ALL), however, one focused only on childhood cancer survivors [32], while the other conducted the search for studies prior to 2013 [26]. Neither of these systematic reviews focuses exclusively on patients with ALL. Here we conduct an in-depth analysis of the available information on ALL in pediatric patients who are under active antineoplastic treatment. It is important to understand the physiological/metabolic changes that occur due to the disease and treatment, which can in turn modify the patients’ nutritional needs and body composition. With this review we aim to assess the effect of different nutrition interventions designed to improve the body composition and nutritional status of children with ALL undergoing antineoplastic oncological treatment.

## Methods

This systematic review was designed and developed based on the Cochrane Collaboration Handbook [34] and reported following the updated PRISMA (Preferred Reporting Items for Systematic Reviews and Meta-Analysis) [35] statement. The protocol was registered and available in the database of the International Prospective Register of Systematic Reviews (PROSPERO: CRD42021266761) [36].

### Inclusion criteria

#### Types of studies

Only randomized controlled trials were included.

#### Types of participants

Studies evaluating patients up to 18 years of age diagnosed with ALL receiving antineoplastic treatment were included. Participants could be male or female, outpatient or hospitalized, and undergoing treatment in public or private hospitals/clinics. Studies including participants over 18 years of age were considered when the population under 18 years of age represented at least 50% of the total sample. Studies that evaluated participants with cancer diagnoses other than ALL were included if at least 50% of the total sample had a diagnosis of ALL.

#### Types of interventions

Interventions including any type of nutritional treatment were considered (e.g., oral, enteral [EN], parenteral [PN], macro and micronutrients, with or without specific supplementation). There was no restriction on the length of the intervention or follow-up. For this review, nutritional support intervention (EN or PN) was defined as the administration of macro and/or micronutrients instead of, or in addition to, normal oral intake [26]. We included vitamins, minerals, and micronutrient supplementation. Normal oral intake was considered when the patients were orally consuming the foods they reach for on a regular basis, i.e., their normal diet.

#### Types of comparisons

No intervention (e.g., standard care, placebo). Studies that compared alternative nutrition interventions were also considered. Examples of interventions that could be compared include nutritional support (EN or PN) vs usual food intake; EN vs PN; usual food intake vs modified diet in macro/micronutrients; and usual intake vs specific nutrient.

#### Types of outcome measures

Primary outcome measures included: fat-free mass (FFM); inflammatory and metabolic markers related to nutritional status (e.g., serum albumin, pre-albumin, C-reactive protein, interleukins, TNF, cytokines); and anthropometric measurements (body circumferences, skinfolds, body mass index [BMI], and body weight). Secondary outcome measures included: fat mass (FM); body water; and adverse events (e.g., frequency, duration, or severity of diarrhea, nausea, vomiting, mucositis, hospitalization days, abnormal biochemical profiles).

Publications in any language were included and there were no date restrictions. Both published and gray literature were included. Studies conducted in any country, including low-, middle- and high-income countries were considered.

### Search strategy

#### The following electronic databases were searched between inception and September 2021

Cochrane Central Register of Controlled Trials (CENTRAL), MEDLINE (Ovid), EMBASE (Ovid), SciELO, and LILACS. Text words and controlled vocabulary were combined in the search strategies and can be found in Supplementary file 1.

#### Supplementary searching included

The detailed supplementary search can be found in the SR protocol [36]. In brief, it included the reference list of included studies, hand searching the conference proceedings of different international organizations, searching research registers for ongoing or unpublished trials, and dissertations & theses.

### Selection of studies

Three review authors (AEGL, JAP, and LRBP) independently performed the screening of the titles and abstracts against the inclusion criteria, and the full text of any potentially relevant record identified by any reviewer was retrieved for closer examination. The inclusion criteria were applied independently against the full text of the selected papers by three reviewers. Three reviewers independently assessed the full texts of the chosen records against the inclusion criteria (AEGL, JAP and LRBP). Discussion and consensus were used to settle disagreements over the studies' eligibility. When doubts remained, third-party arbitration was used (VLT, MMH).

### Data extraction

Three review authors (AEGL, JAP, and LRBP) independently extracted data from each article using a standardized form in Microsoft Excel. The following information was extracted for each trial: characteristics of study (author and year of publication, country and year/s of study intervention, type of study); characteristics of participants (number, age, percentage of males, socioeconomic status, and current cancer treatment); characteristics of the intervention (type of nutrition intervention, description of the intervention, frequency, duration, and doses); outcomes measured; and results.

Review Manager (RevMan 5) [37] was used to analyze quantitative outcome data from the included studies. When necessary, the authors of the studies with missing or unexplained data were contacted. Following the data entry by one review author (AEGL), two additional review authors (JAP and LRBP) independently verified the data entry. Discussion and consensus were used to settle disagreements among review authors on data extraction. Third-party arbitration was applied when necessary (VLT, MMH).

### Assessment of risk of *bias* in included studies

Two independent review authors (AEGL, VLT) evaluated the risk of bias of the included studies using the risk of bias items, as described in the module of Cochrane Childhood Cancer (Module CCG 2014) [38], which are based on the risk of bias domains from the Cochrane Risk of Bias tool [39].

The following items were assessed: adequate sequence generation and allocation concealment (selection bias), masking or blinding of participants and personnel (performance bias), blinding of outcome assessors (detection bias), incomplete outcome data (attrition bias), and selective outcome reporting (reporting bias). Briefly, for the assessment of reporting bias due to selective outcome reporting, when the trial protocol was available, outcomes specified in the protocol and those published in the article were compared to evaluate their similarity. If no protocol was available/published we looked for convincing text in the article that all expected outcomes were reported, including those pre-specified. If no convincing text was found, this domain was rated as unclear risk of bias. All the risk of bias items were evaluated as “low risk of bias”, “high risk of bias”, or “unclear risk of bias”. Disagreements between review authors were resolved by discussion, and third-party arbitration was used when necessary (MMH). Classification of the overall risk of bias for each study was defined as:

- Low risk of bias: studies had mostly low and few unclear risks of bias ratings across all domains.
- Unclear risk of bias: studies had a mix of low, unclear, and high risk of bias ratings across the domains.
- High risk of bias: studies had high risk of bias ratings in several domains, being a critical factor performance bias.

### Strategy for data synthesis

Meta-analysis was conducted where data were reported for the same outcome from at least two studies. Meta-analysis was conducted according to the Cochrane Handbook for Systematic Reviews of Interventions [34]. We assessed the mean difference between groups for continuous outcomes and reported summary estimates with their 95% confidence intervals. We used a random-effects model, which is weighted for both within-study and between-study variation. A narrative overview of the trial results that were unable to be combined for meta-analysis was provided.

Heterogeneity in each meta-analysis was assessed using the I^2^ value, using the categories: low I^2^ (between 0 and 25%), moderate I^2^ (between 25 and 50%), high I^2^ (between 50 and 75%), and very high I^2^ (over 75%) [34]. Constructing funnel plots was our intended method for evaluating non-reporting biases; however, due to the limited number of studies in each meta-analysis, this was not feasible in practice.

## Results

**Fig. 1:**
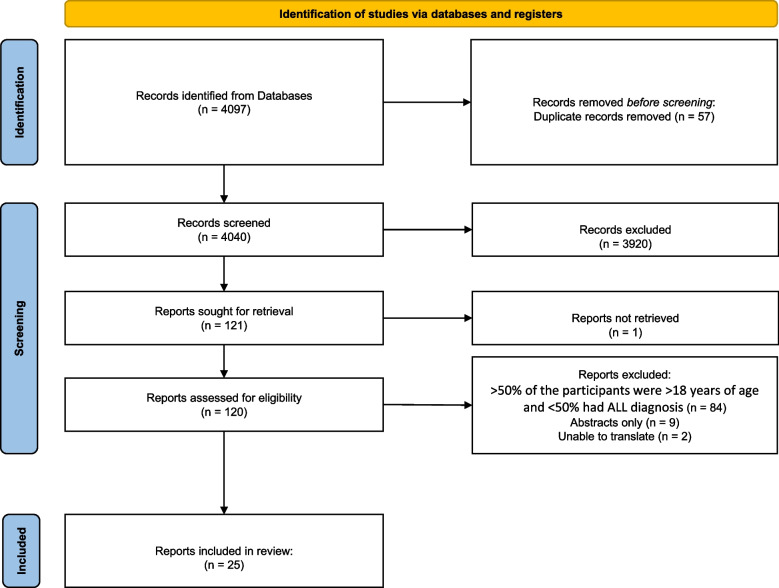
PRISMA flow diagram for the systematic review. ALL, acute lymphoblastic leukemia

We identified 4040 records after removal of duplicates (Fig. 1). Of these, 3920 were disqualified after the first screening (titles and abstracts). The full text of 121 records were evaluated for eligibility, of which 95 records were excluded for not meeting the inclusion criteria and one could not be retrieved (Supplementary file 2). In total, 25 studies met the criteria and were included in this review [40–64] (Fig. 1).

Table S1 shows the characteristics of the included studies, which were all randomized controlled trials. Six studies were conducted in Egypt [40, 51, 55, 56, 59, 63], four in the USA [46, 47, 54, 62], three in Brazil [50, 52, 53], two in each of Iran [41, 43], Turkey [42, 44], and Spain [57, 58], and one in each of Indonesia [48], China [49], Malaysia [45], Denmark [60], Venezuela [61], and Mexico [64]. The following nutrition interventions were tested, Compound/Food: black seed oil (*n* = 2) [55, 59], glutamine(*n* = 4) [46, 48, 49, 62], honey (*n* = 2) [40, 56], probiotics (*n* = 1) [64], soy nut powder (*n* = 1) [41], ω-3 (*n* = 3) [45, 51, 57], and whey protein hydrolysate (*n* = 1) [60]; Micronutrient: selenium (*n* = 2) [50, 53], vitamin A (*n* = 1) [42], vitamin D (*n* = 2) [44, 54], vitamin E (*n* = 2) [43, 63], and zinc (*n* = 1) [52]; Nutritional support: enteral nutrition (*n* = 1) [61], individualized nutritional counseling (*n* = 1) [47], and parenteral nutrition (*n* = 1) [58].

### Outcomes measured

The relevant outcomes (for this review) reported in each trial are presented in Table S1. In relation to our three primary outcomes, none of the included studies reported fat-free mass as an outcome. Inflammatory and metabolic markers related to nutritional status were reported in many studies but varied greatly across studies and interventions. The most frequently reported markers were hemoglobin (Hb) [40, 41, 43, 65], serum albumin [43, 49, 58, 59], alkaline phosphatase [44, 51, 59], and calcium and phosphorus, which were measured in two trials using vitamin D supplementation [44, 54]. For our primary outcome of anthropometric measurements, five studies reported body weight [41, 45, 49, 52, 61], three reported body mass index [41, 47, 61], and waist circumference [41, 47] and mid-upper arm circumference [45, 61] were each reported in two trials; triceps skinfold thickness was reported in one trial [49].

For our secondary outcomes, fat mass or total body water were not reported as an outcome in any of the studies. However, a range of different adverse events were reported, including febrile neutropenia [50, 56, 60], days of hospitalization [48, 62], and chemotherapy complications [41, 46, 60, 64].

### Risk of *bias* in included studies

**Fig. 2:**
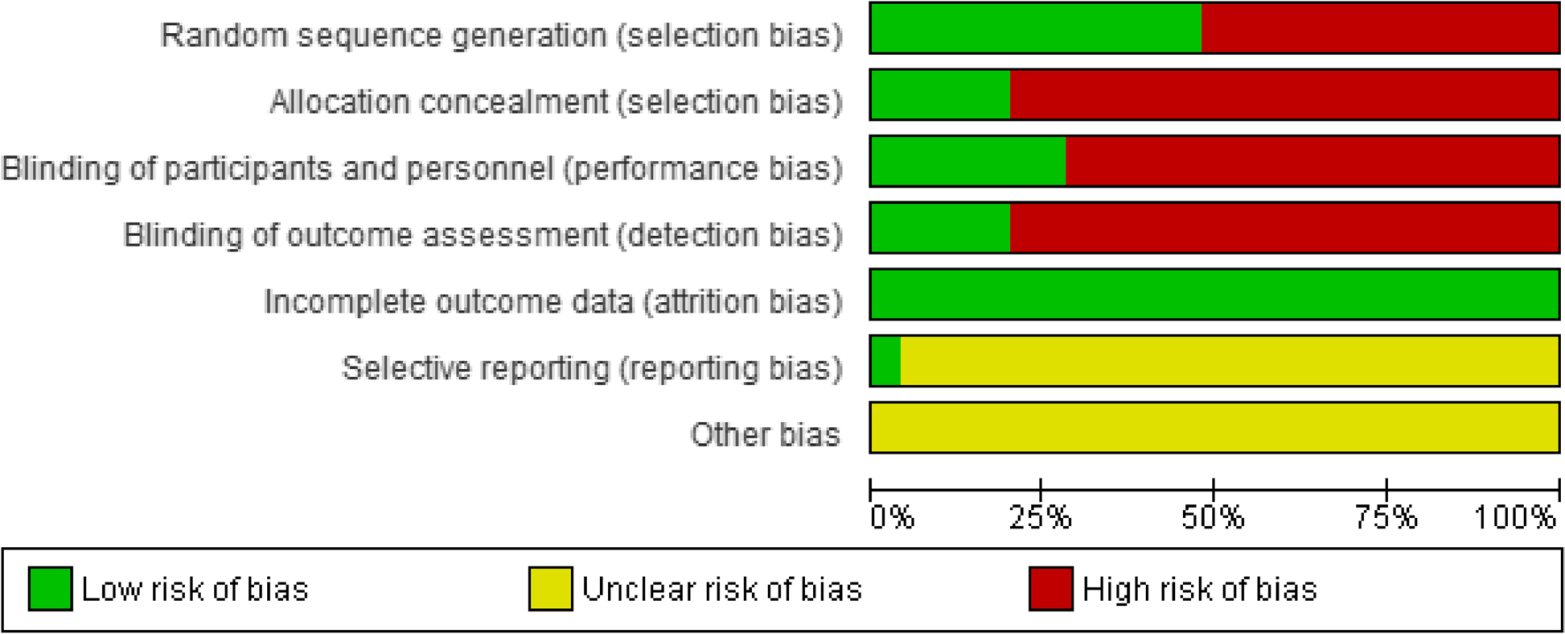
Risk of bias graph. Judgements about each risk of bias item presented as percentages across all included studies. In the x axis are the percentages, and the y axis shows the different types of bias. In green are classified the studies that meet the criteria for a low risk of bias, in yellow those with an unclear risk of bias, and in red the ones with high risk of bias

The assessments of risk of bias for each study are presented in Figure S1 and summarized in Fig. 2. Of the 25 included studies two [60, 41] were classified as low risk of bias, six [46, 40, 48, 51, 52, 56] as unclear risk of bias, and 17 as high risk of bias [40, 42–45, 47, 49, 50, 53–55, 57–59, 61–64].The lack of allocation concealment (20 studies) [40, 42–50, 52–54, 57–59, 61–64] and lack of blinding of outcome assessors (20 studies) [40, 42–45, 47, 49–51, 53–59, 61–64] were the main limitations, followed by the lack of blinding (participants and personnel) (18 studies) [40, 42–45, 47, 49, 53–59, 61–64] and random sequence generation (13 studies) [42, 44–46, 48, 50, 52, 53, 57–59, 61, 63].

### Effects of interventions

The results of the included studies for each intervention are reported in Table 1. All findings, except where otherwise indicated, should be interpreted cautiously because most of the included studies have a high risk of bias.

**Table 1:** Results of included studies

Author, year	Country and years of study	Overall risk of bias	Main findings
***Compound/Food***			
**Black seed oil**			
Hagag, 2015 [59]	Egypt 2010–2014	High risk	ALT (UI/L): 57.1 ± 6.53 vs 103.6 ± 24.39, *P* = 0.000 AST (UI/L): 59.9 ± 25.03 vs 99.85 ± 17.43, *P* = 0.000 ALP (UI/L): 220.85 ± 25.03 vs 482.8 ± 29.47, *P* = 0.000 Total serum protein (g/dL): *P*˃0.05 Serum albumin (mg/dL): *P*˃0.05 Total bilirubin (mg/dL): 0.83 ± 0.14 vs 2.21 ± 0.83, *P* = 0.000
Hagag, 2020 [55]	Egypt 2016–2018	High risk	Creatinine (mg/dL): *P*˃0.05 Urea (mg/dL): *P*˃0.05 BUN (mg/dL): *P*˃0.05
**Glutamine**			
Aquino, 2005 [62]	USA 1998–2002	High risk	Days of intravenous narcotic use: 12.1 ± 1.5 vs 19.3 ± 2.8, *P* = 0.03 Days of TOTAL PN use: 17.3 ± 1.7 vs 27.3 ± 3.6, *P* = 0.01 Episodes of patients who developed bacteremia: *P*˃0.05 Hospital days: *P*˃0.05
Han, 2016 [49]	China 2013–2014	High risk	Weight (Kg): *P*˃0.05Triceps skinfold (mm): *P*˃0.05 Serum albumin (g/L): (32.57 ± 3.05 vs 27.15 ± 3.29), *P* < 0.05 Serum pre-albumin (mg/L): *P*˃0.05 Retinol binding protein (mg/L): 24.59 ± 5.3 vs 19.52 ± 2.49, *P* < 0.05 Presence of edema: 6 vs 16. *P* < 0.05
Sands, 2017 [46]	USA Not reported	Unclear risk	Presence of sensory neuropathy: 11 vs 19, *P* = 0.02 Presence of motor neuropathy: *P* = 0.559 Side effects: *P*˃0.05, NS
Widjaja, 2020 [48]	Indonesia Not reported	Unclear risk	Occurrence of oral mucositis: 4.2% vs 62.5%, *P* = 0.001 Hospital days: 7.67 ± 0.59 vs 12 ± 2.57, *P* = 0.005
**Honey**			
Abdulrhman, 2012 [56]	Egypt 2010–2011	Unclear risk	Recovery time: 4.25 ± 1.25 vs 6.1 ± 2.47, *P* = 0.0005
Abdulrhman, 2016 [40]	Egypt 2011–2013	High risk	Hemoglobin (g/dL): 11.3 ± 1.23 vs 8.57 ± 1.14, *P* < 0.001 Episodes of febrile neutropenia: *P* = 0.131 Number (%) patients who developed febrile neutropenia: 22% vs 45%, *P* = 0.00004 Hospital days: *P* = 0.126
**Probiotics**			
Reyna-Figueroa, 2019 [64]	Mexico Not reported	High risk	Constipation: RR 0.4 (0.2–0.6), *P* < 0.05 Abdominal distention: RR 0.4 (0.2–0.7), *P* < 0.05 Meteorism: RR 0.5 (0.4–0.7), *P* < 0.05 Diarrhea: RR 0.5 (0.2–1.2), *P* < 0.05 Vomiting: RR 0.4 (0.2–0.7), *P* < 0.05 Dyspepsia: RR 0.6 (0.3–1.2), *P* < 0.05 Nausea: RR 0.5 (0.4–0.8), *P* < 0.05
**Soy nut powder**			
Ramezani, 2018 [41]	Iran 2016–2017	Low risk	Weight (Kg): 22.4 ± 7 vs 18.8 ± 6.4, *P* = 0.001 BMI (Kg/m^2^): 16.5 ± 2 vs 15.2 ± 1.8, *P* = 0.001 Hemoglobin (g/dL): 11.4 ± 1.6 vs 10.4 ± 1.6, *P* = 0.001 Waist circumference (cm): *P* = 0.616 Pain: *P* = 0.065 Fatigue: 5 (3.5, 7.5) vs 6 (3.4, 8.0), *P* = 0.041, NS Nausea: *P* = 0.267 Depression: *P* = 0.220 Anxiety: *P* = 0.800 Drowsiness: *P* = 0.319 Appetite: *P* = 0.535 Well-being: *P* = 0.509 Dyspnea: *P* = 0.817
**ω-3**			
Abu Zaid, 2012 [45]	Malaysia 2005	High risk	Weight (Kg): *P*˃0.05 MUAC (cm): 6.29 ± 1 vs 5.16 ± 0.89, *P* = 0.001
Baena-Gómez, 2013 [57]	Spain Not reported	High risk	Cholesterol (mg/dL): *P*˃0.05 Triglycerides (mg/dL): *P*˃0.05 HDL (mg/dL): *P*˃0.05 LDL (mg/dL): *P*˃0.05
Elbarbary, 2016 [51]	Egypt Not reported	Low risk	Total bilirubin (mg/dL): 0.91 ± 0.4 vs 3.9 ± 1.4, *P* < 0.001 ALP (UI/L): 189.6 ± 18.7 vs 519.6 ± 34.3, *P* < 0.001 ALT (UI/L): 27.8 ± 10.1 vs 69.8 ± 10.6, *P* < 0.001 AST (UI/L): 40.6 ± 11.2 vs 69.8 ± 10.6, *P* < 0.001
**Whey protein hydrolysate**			
Rathe, 2019 [60]	Denmark 2013–2016	Low risk	Patients with febrile neutropenia: *P*˃0.05 Days with febrile neutropenia *P*˃0.05 Oral mucositis: 48.28% vs 64.52%, *P* = 0.02 Abdominal pain: *P*˃0.05 Diarrhea: *P*˃0.05
***Micronutrient***			
**Selenium**			
Vieira, 2014 [53]	Brazil 2010–2012	High risk	"There were no significant alterations in the analyzed parameters (fatigue, nausea, appetite loss, physical function) when the data from the beginning of the treatment were compared with those obtained after supplementation with Selenium and the use of placebo"
Rocha, 2016 [50]	Brazil Not reported	High risk	Hemoglobin (g/dL): *P*˃0.05 Febrile neutropenia cases: "During the analyzed period, Se supplementation was able to minimize the triggering of febrile neutropenia cases (characterized by counts equal to or < 500 neutrophils/mL). Neutropenia was observed in 1 patient at the beginning, 1 patient during Se supplementation, and 3 patients during supplementation with placebo. There was a significant increase in the number of circulating neutrophils during supplementation: from 20 neutrophils/mL (beginning of the study) to 320 neutrophils/mL (supplementation with Se)"
**Vitamin A**			
Dagdemir, 2004 [42]	Turkey 2000–2004	High risk	Gastrointestinal toxicity grade: *P*˃0.05 Hematological toxicity grade: *P*˃0.05 Skin toxicity grade: *P*˃0.05 Systemic toxicity grade: *P*˃0.05
**Vitamin D**			
Orgel, 2017 [54]	USA 2011–2014	High risk	Corrected Ca (mg/dL): *P*˃0.05 Phosphorus (mg/dL): *P*˃0.05 Vitamin D (ng/mL): 26.5 ± 12.4 vs 19 ± 7.4, NR
Solmaz, 2021 [44]	Turkey 2011–2012	High risk	Ca (mg/dL): *P*˃0.05 P (mg/dL): *P*˃0.05 Mg (mg/dL): *P*˃0.05 ALP (u/L): *P*˃0.05
**Vitamin E**			
Al-Tonbary, 2009 [63]	Egypt 2006–2007	High risk	Hematological complications N (%): 40% vs 100%, *P* = 0.001
Bordbar, 2018 [43]	Iran 2014–2015	High risk	“Hemoglobin (g/dL), Serum protein (g/dL), Serum albumin (g/L): "At the end of the study, no statistically significant differences were found between groups"
**Zinc**			
Consolo, 2013 [52]	Brazil 2010–2012	Unclear risk	Weight gain (Kg): + 2000 g vs + 100 g, *P* = 0.032 Presence of oral Mucositis: *P* = 0.923
***Nutritional support***			
**Enteral Nutrition**			
Noguera, 2005 [61]	Venezuela 2010	High risk	Weight (kg): *P*˃0.05 MUAC (cm): *P*˃0.05 BMI (Kg/m^2^): *P*˃0.05
**Individualized nutritional counseling**			
Li, 2016 [47]	USA Not reported	High risk	BMI (Kg/m^2^): *P*˃0.05 Waist circumference (cm): *P*˃0.05
**Parenteral nutrition**			
Jiménez, 1999 [58]	Spain Not reported	High risk	Number of infections: *P*˃0.05 Serum prealbumin (g/L): *P*˃0.05 Serum albumin (g/dL): *P*˃0.05 Transferrin (mg/dL): *P*˃0.05 Retinol binding protein (mg/L): *P*˃0.05 Serum cholesterol (mg/dL): *P*˃0.05 HDL (mg/dL): 22.6 ± 12.6 vs 15.4 ± 9.6, *P* < 0.05 LDL (mg/dL): *P*˃0.05 Triglycerides (mg/dL): *P*˃0.05

*ALL* Acute lymphoblastic leukemia, *ALP* Alkaline phosphatase, *ALT* Alanine aminotransferase, *AST* Aspartate aminotransferase, *BMI* Body mass index, *BUN* Blood urea nitrogen, *Ca* Calcium, *DHA* Docosahexaenoic acid, *DRI* Dietary reference intakes, *DOX* Doxorubicin, *EPA* Eicosapentaenoic acid, *HDL* High-density lipoprotein, *HDMTX* High-dose methotrexate, *LCPUFAS* Long-chain polyunsaturated fatty acids, *LDL* Low-density lipoprotein, *LCT* Long-chain triglycerides, *MCT* Mid-chain triglycerides, *Mg* Magnesium, *MUAC* Mid-upper arm circumference, *NAC* N-acetylcysteine, *NS* No significative difference, *RR* Relative risk, *P* phosphorus

#### Glutamine

The use of supplementation with glutamine was assessed in four studies [46, 48, 49, 62] over a range of doses (Table S1). The risk of bias was low for one study [62], one had a high risk of bias [49], and two had an unclear risk of bias [46, 48]. Only one study with high risk of bias measured our primary outcomes of interest [49]. They compared the daily use of glutamine-enriched nutritional therapy over the treatment course versus a non-glutamine control group. Results showed a significant increase in serum albumin (*P < *0.05) and retinol binding protein (*P < *0.05) concentration.

All four studies assessed our secondary outcomes of adverse events but using a variety of measures (e.g., bacteremia, edema, and oral mucositis). In general, authors found fewer adverse events in the glutamine intervention group (Table 1). Only two studies [48, 62] measured the same adverse event outcome (days in hospital). The random effects meta-analysis found a combined reduction of 3.44 (95% CI: 1.79 – 5.10) days in the hospital for the glutamine group, but with very high heterogeneity (I^2^ = 85%) (Figure S2), which implies that the result and its applicability in clinical practice should be interpreted cautiously.

#### Honey

Two studies tested the use of different doses of honey versus a control group (Table S1) [40, 56]. Only one study with a high risk of bias measured one of our primary outcomes (i.e., hemoglobin [40], here they tested a dose of honey twice weekly for 12 weeks and found an increase in serum hemoglobin (*P* < 0.001).

Both studies measured the secondary outcomes of recovery time, febrile neutropenia episodes, number of patients who developed febrile neutropenia, and number of days in hospital. In general, they found fewer adverse events when honey was consumed compared to the control group (Table 1). These results should be treated with caution due to the high [40] and unclear risk of bias [56].

#### Soy nuts

One study [41] with a low risk of bias tested powdered soy nuts by recommending patients consume one sachet (30 g) alongside their food every day for 12 weeks (Table S1). They measured the primary outcomes of body weight (Kg), BMI, and hemoglobin, and found a significant increase in all of them at the end of the intervention (Table 1), which is a positive outcome of the intervention since many patients started with undernutrition.

This study also measured the secondary outcome of adverse events but using a variety of measures (e.g., pain, fatigue, and nausea). In general, no differences were observed at the end of the intervention.

#### Vitamin A

One study [42] with a high risk of bias tested a single high-dose vitamin A (180,000 IU, 54 mg) supplementation 24 h before a high dose of methotrexate was provided (Table S1). They only measure the secondary outcome of gastrointestinal, hematological, skin, and systemic toxicity. No differences were found between the intervention and control group (Table 1).

#### Whey protein hydrolysate

One study [60] tested the use of whey protein hydrolysate and bovine colostrum in sachets. The number of sachets was defined according to the patient’s body weight (Table S1). This study had a low risk of bias. They did not measure any of our primary outcomes but measured some of our secondary outcomes such as: days with febrile neutropenia, number of patients with febrile neutropenia, abdominal pain, and diarrhea. No differences were observed in these secondary outcomes; however, the presence of oral mucositis showed a significant reduction between the intervention and the control group (48.28% vs 64.52%, *P* = 0.02) (Table 1).

#### Vitamin E

Two studies [43, 63] with a high risk of bias tested vitamin E, each using a different supplement dose (Table S1). Only one of the studies measured one of our primary outcomes of interest [43]. The authors tested a daily oral dose of vitamin E along with their routine chemotherapy drugs; one study [63] measured the secondary outcome of hematological complications, finding fewer events (40% vs 100%, *P* = 0.001) in the intervention group (Table 1).

#### Vitamin D

Two studies [44, 54] with a high risk of bias tested vitamin D using different doses (Table S1). Both studies measured primary outcomes of interest (i.e., inflammatory and metabolic markers related to nutritional status).

Solmaz *et. al*. [44], tested a single oral dose of vitamin D3 on day one of chemotherapy. No change was observed in serum levels of Ca, P, Mg, or alkaline phosphatase (Table 1). Similar results were observed by Orgel *et. al*. [54], with no differences in serum values of Ca, P, and Vitamin D (Table 1).

Both studies measured the same primary outcomes (Ca and P), but the meta-analysis did not show significant differences between the intervention and control groups (Ca: 0.11 mg/dL, 95% CI: -0.24 – 0.46; P: -0.26 mg/dL, 95% CI: -0.66 – 0.14) (Figures S3 and S4).

#### ω-3

Three studies [45, 51, 57] tested ω-3 using different doses (Table S1). One study had a low risk of bias [51], and the other two had a high risk of bias [45, 57]. All three studies measured different primary outcomes. Elbarbary *et. al*. [51], found a significant improvement in the levels of alkaline phosphatase, alanine aminotransferase, aspartate aminotransferase, and total bilirubin (Table 1). Results by Abu Zaid *et. al*. [45], showed no differences in body weight after the intervention; however, mid-upper arm circumference values improved significantly. No differences were found by Baena-Gómez *et. al*. [57], in any of the variables analyzed (Table 1).

#### Individualized nutrition intervention

One study[47] with a high risk of bias measured our primary outcomes of BMI and waist circumference. They tested an individualized nutrition counseling intervention (guided by a certified dietitian) compared to a standard care group (nutrition handouts by request, physician referral, or when nutritional risk was diagnosed) for a total of 12 monthly follow-up sessions (Table S1). No differences were observed between groups at the end of the intervention (Table 1).

#### Selenium

Two studies [50, 53] with a high risk of bias tested the use of different selenium doses. One study [50] measured the primary outcome hemoglobin but found no difference between groups. Both studies [50, 53] measured different secondary adverse events outcomes, however, no significant differences were observed in any of the analyzed parameters (Table 1).

#### Zinc

One study [52] with a high risk of bias tested the use of a syrup containing zinc in the form of a chelate solution divided into two doses (Table S1). They found no significant difference in body weight gain and the presence of oral mucositis between groups (Table 1).

#### Black seed oil

Two studies with a high risk of bias tested black seed oil [55, 59]. Both studies measured one of the primary outcomes of interest (serum biomarkers). Hagag *et. al*. [59], found significant reductions in alkaline phosphatase, alanine aminotransferase, aspartate aminotransferase and total bilirubin, but no differences in total serum protein and albumin (Table 1). Hagag et. al. [55], did not find differences in creatinine, urea, or blood urea nitrogen levels at the end of the study.

#### Parenteral nutrition

One study [58] with a high risk of bias tested a combination of nutrients using parenteral nutrition (Table S1). Although, Jiménez et. al., (49) assessed many of our primary outcomes of interest, only high-density lipoprotein cholesterol increased significantly post-intervention, showing a positive effect of the intervention (Table 1). One secondary adverse event outcome of interest (number of infections) was measured in this study, but no difference was found (Table 1).

#### Enteral nutrition

One study [61] with a high risk of bias utilized an enteral formula that represented 30% of the individual’s caloric requirement (Table S1). They measured the primary outcomes of interest weight, mid-upper arm circumference, and BMI, but no differences were found between groups (Table 1).

#### Probiotics

One study [64] with a high risk of bias tested an oral probiotic supplementation with *Lactobacillus rhamnosus* (one sachet) twice a day during the intervention period (see Table S1). They did not measure any of our primary outcomes, but they assessed the following secondary adverse event outcomes: constipation, abdominal distention, meteorism, diarrhea, vomiting, dyspepsia, and nausea, observing a significant relative risk reduction in all of the above-mentioned variables (Table 1).

## Discussion and conclusion

To the best of our knowledge, this represents the first systematic review of randomized controlled trials examining the impact of nutrition interventions (Table S1) on pediatric oncology patients diagnosed with ALL while undergoing active antineoplastic treatment. Our review included twenty-five randomized controlled trials, each testing different interventions and outcome variables; consequently, we were not able to pool most of the results into a meta-analysis. None of the interventions showed consistent evidence of a positive effect on the children’s nutritional status. However, there were indications that hospitalization days, presence of edema and neuropathy, recovery time, hemoglobin, and gastrointestinal side effects might be improved using glutamine, honey, black seed oil, and probiotics (Table 1), but the high risk of bias of the included trials limits the conclusions that can be made.

Although some of our primary outcomes of interest were not measured in the studies included in this systematic review (e.g. fat free mass, limited reporting of anthropometric measures), changes in body composition associated with pediatric ALL treatment have been reported in cohort and cross-sectional studies [5, 8, 10, 12, 27, 28, 66, 67] (changes in bone density, fat- and fat-free mass and total body water). Unfortunately to our knowledge, and as shown in this review, there are no published RCTs assessing the effect of nutritional interventions on changes in body composition of patients diagnosed with ALL. While the evidence suggest that it is essential to recognize and manage changes in body composition to improve treatment results in patients with ALL, the best way to do this remains unclear. This calls for the use of interdisciplinary cooperation, studies with larger sample sizes, longitudinal designs, and standardized measurement instruments. Such efforts can support professional decision-making, providing a deeper understanding of how therapy affects multiple aspects of children's life, and contribute to developing focused interventions that improve results.

The heterogeneity of the treatments tested in the trials impacts the applicability and interpretation of this systematic review [34, 39]. The presence of diverse treatment regimens and outcome measures across studies makes it challenging to directly compare the interventions for specific outcomes, making it difficult to draw uniform conclusions. The wide range of treatments also limits the interpretation and generalizability of the findings for clinical practice.

Interventions using the nutrients analyzed in this review have been reported in the literature as having beneficial effects for patients of different ages or with other types of cancer [68–73]. It is important to note that ALL is a complex disease, with different stages and pharmacological treatments that, in turn, interact with the individuals’ response [7, 24, 74–79]; all these factors could influence and hinder the effect of the interventions evaluated in this review.

Previous reviews [26, 32] of nutritional interventions in pediatric oncology have consistently highlighted the variability in clinical outcome measures reported. Recent research [14, 21, 26, 32, 75, 80] and our review emphasize the need to determine the most useful outcome measures for pediatric oncology trials (since the physiological/metabolic changes that occur due to the disease and treatment could modify the patient’s clinical response, nutritional needs, and body composition). As a starting point, we recommend evaluating and reporting the variables suggested by the Pan American Health Organization [81] related to nutritional status, among which are the Nutrition screening tools (questionnaires), anthropometric measurements (BMI, triceps skinfold, mid-upper arm and waist circumference), biochemistry exams (liver and renal function test, lipid and glucose panel, serum concentration proteins, and micronutrients), dietary intake (macro- and micro-nutrient intake and dietary patterns) and when possible body composition (bioelectrical impedance analysis and dual x-ray absorptiometry).

Discussion and consensus among a multidisciplinary and globally representative group of experts is needed to determine which of these should be prioritized for measurement in trials. In addition to determining the outcomes of clinical interest in ALL patients [82], research is needed to determine, and measure, the outcomes that are important to the patients and their families (e.g. quality of life) [83–86]. This will contribute to the development of clinical practice guidelines that follow international standards [87].

Strengths of this systematic review include the high-quality methods, including an a priori protocol, and use of three reviewers for independent screening and selection of studies, and for data extraction. Also, the review includes the use of two reviewers for risk of bias assessment. Although randomized controlled trials constitute the best evidence for interventions [34, 88], our review was limited by the small number of trials available for each intervention tested, and the high risk of bias of most of the included trials. The heterogeneity in the interventions and outcome variables measured also prevented our ability to make conclusions. A further limitation of our review was that patients and their families were not involved in the development and interpretation of this review.

We recommend that researchers undertaking trials in this area take greater care to ensure (and report) allocation concealment, and adequate randomization.

Utilizing centralized randomization techniques like pharmacy-controlled randomization, sequentially numbered, opaque, sealed envelopes, or computer-generated randomization codes is recommended by Cochrane [34, 39] to ensure the allocation sequence is hidden from individuals who are active in participant recruitment [34, 39].

Also, the blinding techniques suggested by Cochrane can reduce the risk of bias of the studies [34, 39]. Some suggested strategies are the blinding of the participants, healthcare professionals who participate in delivering the intervention, and outcome assessors to the treatment assignment of participants [34, 39]. Finally, consensus among researchers and practitioners in this area as to what outcomes are most important to measure and report would also be helpful to ensure the usefulness of future research [5, 72, 81, 82, 84, 89–91].

Future RCT studies should use international reporting guidelines such as the Standard Protocol Items: Recommendations for Interventional Trials (SPIRIT) [92, 93] and the Consolidated Standards of Reporting Trials (CONSORT) [94, 95], for the development of the protocol and the reporting of results, respectively. Finally, consensus among researchers and practitioners in this area as to what outcomes are most important to measure and report would also be helpful to ensure the usefulness of future research.

In conclusion, nutritional interventions are a complex issue that require careful consideration and individualized treatment plans. While some supplements may have potential health benefits, for children with cancer, maintaining and/or achieving optimal nutritional status requires an emphasis on serving balanced, healthy meals. In addition, a nutritional intervention that considers the use of supplementation should only be considered when the physician or registered dietitian considers that there is a risk–benefit relationship that helps the patient.

Although with the interventions analyzed in this review it is not possible to provide a definitive answer to the research question, this work highlights the need for further research in nutrition interventions, specifically in pediatric patients with ALL undergoing active antineoplastic treatment. High quality randomized trials that measure the most important outcomes are needed to add to the evidence base and help clinicians make the best possible decisions to improve the health and quality of life of their patients.

### Supplementary Information

Additional file 1. Search terms and strategy.

Additional file 2. List of excluded studies.

Additional file 3. PRISMA Checklist.

Additional file 4. (Table S1).

Additional file 5. Figure S1. Risk of bias summary. Judgements about each risk of bias item for each included study. In the x axis are the 25 studies that met the inclusion criteria, and the y axis shows the different types of bias. In green are classified the studies that meet the criteria for a low risk of bias, in yellow those with an unclear risk of bias, and in red the ones with high risk of bias. Figure S2. Forest plot showing the effect of glutamine vs placebo on hospitalization days. Figure S3. Forest plot showing the effect of vitamin D supplementation vs placebo on serum calcium (Ca). Figure S4. Forest plot showing the effect of vitamin D supplementation vs placebo on serum phosphorus (P).

Additional file 6.

## Data Availability

Data is provided within the manuscript or supplementary information files.

## Abbreviations

ALL: Acute lymphoblastic leukemia
EN: Enteral nutrition
FFM: Fat-free mass
FM: Fat mass
PN: Parenteral nutrition

## References

[1] American Cancer Society. Childhood and Adolescent Blood Cancer Facts and Statistics | Leukemia and Lymphoma Society. Cancer Facts & Figures 2021. Published online 2021:1–2.https://www.lls.org/facts-and-statistics/childhood-and-adolescent-blood-cancer-facts-and-statistics%0Ahttps://www.cancer.org/research/cancer-facts-statistics.html

[2] Kristina SA, Endarti D, Aditama H, American Cancer Society (2018). Global cancer - Facts&Figures 4th edition. Am Cancer Soc..

[3] American Cancer Society. About Acute Lymphocytic Leukemia (ALL). Accessed March 1, 2021. https://www.cancer.org/cancer/acute-lymphocytic-leukemia/about.html

[4] PDQ® Pediatric Treatment Editorial Board. PDQ Childhood Acute Lymphoblastic Leukemia Treatment. National Cancer Institute: PDQ Cancer Information Summaries. Published online 2021:1–176. http://www.ncbi.nlm.nih.gov/pubmed/26389206

[5] Murphy-Alford AJ, Prasad M, Slone J, Stein K, Mosby TT (2020). Perspective: Creating the Evidence Base for Nutritional Support in Childhood Cancer in Low- and Middle-Income Countries: Priorities for Body Composition Research. Adv Nutr.

[6] Prado CM, Purcell SA, Laviano A (2020). Nutrition interventions to treat low muscle mass in cancer. J Cachexia Sarcopenia Muscle.

[7] Murphy AJ, Wells JC, Williams JE, Fewtrell MS, Davies PS, Webb DK. Body Composition in Children in Remission from Acute Lymphoblastic Leukemia 1–3. Vol 83.; 2006. https://academic.oup.com/ajcn/article/83/1/70/4649610 10.1093/ajcn/83.1.70 16400052

[8] Yang HR, Choi HS (2019). A prospective study on changes in body composition and fat percentage during the first year of cancer treatment in children. Nutr Res Pract.

[9] Brinksma A, Roodbol PF, Sulkers E (2015). Changes in nutritional status in childhood cancer patients: A prospective cohort study. Clin Nutr.

[10] Chinceşan MI, MǍrginean CO, VoidǍzan S, Ioana Chinces M, Oana Ma C, Voida S (2016). Assessment of body composition in a group of pediatric patients with cancer: a single Romanian center experience. J Pediatr Hematol Oncol..

[11] Ladas EJ, Orjuela M, Stevenson K (2016). Dietary intake and childhood leukemia: The Diet and Acute Lymphoblastic Leukemia Treatment (DALLT) cohort study. Nutrition.

[12] Murphy AJ, White M, Davies PSW (2010). Body composition of children with cancer. Am J Clin Nutr.

[13] Ramos Chaves M, Boléo-Tomé C, Monteiro-Grillo I, Camilo M, Ravasco P (2010). The diversity of nutritional status in cancer: new insights. Oncologist.

[14] Barr RD, Stevens MCG (2020). The influence of nutrition on clinical outcomes in children with cancer. Pediatr Blood Cancer.

[15] Arends J, Bachmann P, Baracos V (2017). ESPEN guidelines on nutrition in cancer patients. Clin Nutr.

[16] Beer SS, Juarez MD, Vega MW, Canada NL (2015). Pediatric malnutrition: putting the new definition and standards into practice. Nutr Clin Pract.

[17] Den Hoed MAH, Pluijm SMF, De Groot-Kruseman HA (2015). The negative impact of being underweight and weight loss on survival of children with acute lymphoblastic leukemia. Haematologica.

[18] Villanueva G, Blanco J, Rivas S, et al. Nutritional status at diagnosis of cancer in children and adolescents in Guatemala and its relationship to socioeconomic disadvantage: A retrospective cohort study. Pediatr Blood Cancer. 2019;66(6). 10.1002/pbc.27647 10.1002/pbc.27647 30729661

[19] Arends J, Baracos V, Bertz H (2017). ESPEN expert group recommendations for action against cancer-related malnutrition. Clin Nutr.

[20] Organización Mundial de la Salud (OMS). Malnutrición. Accessed January 21, 2021. https://www.who.int/es/news-room/fact-sheets/detail/malnutrition

[21] Rogers PC, Barr RD. The relevance of nutrition to pediatric oncology: A cancer control perspective. Pediatr Blood Cancer. 2020;67(S3). 10.1002/pbc.28213 10.1002/pbc.28213 32096351

[22] Martín-Trejo JA, Núñez-Enríquez JC, Fajardo-Gutiérrez A (2017). Early mortality in children with acute lymphoblastic leukemia in a developing country: the role of malnutrition at diagnosis. A multicenter cohort MIGICCL study. Leuk Lymphoma..

[23] Loeffen EAH, Brinksma A, Miedema KGE, de Bock GH, Tissing WJE (2015). Clinical implications of malnutrition in childhood cancer patients—infections and mortality. Support Care Cancer.

[24] El KS, Omar M (2020). Nutritional considerations in childhood acute lymphoblastic leukemia. Cancer Oncol Res..

[25] Mehta NM, Corkins MR, Lyman B (2013). Defining pediatric malnutrition: A paradigm shift toward etiology-related definitions. J Parenter Enter Nutr.

[26] Ward EJ, Henry LM, Friend AJ, Wilkins S, Phillips RS. Nutritional support in children and young people with cancer undergoing chemotherapy. Cochrane Database System Rev. 2015;2015(8). 10.1002/14651858.CD003298.pub3 10.1002/14651858.CD003298.pub3 PMC8752126 26301790

[27] Bradley KT, Westlund NK (2017). The importance of body composition in explaining the overweight paradox in cancer. J Neruosci Res.

[28] Murphy AJ, White M, Elliott SA, Lockwood L, Hallahan A, Davies PSW (2015). Body composition of children with cancer during treatment and in survivorship. Am J Clin Nutr.

[29] Morrell MBG, Baker R, Johnson A, Santizo R, Liu D, Moody K. Dietary intake and micronutrient deficiency in children with cancer. Pediatr Blood Cancer. 2019;66(10). 10.1002/pbc.27895 10.1002/pbc.27895 PMC6707843 31286672

[30] Ladas EJ, Arora B, Howard SC, Rogers PC, Mosby TT, Barr RD (2016). A Framework for Adapted Nutritional Therapy for Children With Cancer in Low- and Middle-Income Countries: A Report From the SIOP PODC Nutrition Working Group. Pediatr Blood Cancer.

[31] Kuiken NSS, Rings EHHM, van den Heuvel-Eibrink MM, van de Wetering MD, Tissing WJE (2017). Feeding strategies in pediatric cancer patients with gastrointestinal mucositis: a multicenter prospective observational study and international survey. Support Care Cancer.

[32] Cohen JE, Wakefield CE, Cohn RJ. Nutritional interventions for survivors of childhood cancer. Cochrane Database System Rev. 2016;2016(8). 10.1002/14651858.CD009678.pub2 10.1002/14651858.CD009678.pub2 PMC6486279 27545902

[33] Ladas EJ, Sacks N, Meacham L, et al. Invited Review A Multidisciplinary Review of Nutrition Considerations in the Pediatric Oncology Population: A Perspective From Children’s Oncology Group. Nutr Clin Pract. 2005;20:377–93. 10.1177/0115426505020004377 16207678

[34] Higgins JPT, Thomas J, Chandler J, Cumpston M, Li T, Page MJ, Welch VA (editors). Cochrane Handbook for Systematic Reviews of Interventions version 6.4 (updated August 2023). Cochrane. 2023. Available from www.training.cochrane.org/handbook.

[35] Page MJ, McKenzie JE, Bossuyt PM, The PRISMA (2020). statement: An updated guideline for reporting systematic reviews. The BMJ.

[36] Guzman Leon AE, Lopez-Teros V, Avila-Prado J, Bracamontes-Picos L, Haby MM, Stein K. Protocol for a Systematic Review: Nutritional interventions in children with acute lymphoblastic leukemia undergoing antineoplastic treatment. International prospective register of systematic reviews. 2021;PROSPERO(CRD42021266761). 10.1186/s40795-024-00892-4 PMC11186292 38898513

[37] Copenhagen: The Nordic Cochrane Centre TCC. Review Manager (RevMan) (2014) Version 5.3. Accessed December 4, 2023. https://www.scirp.org/(S(czeh2tfqw2orz553k1w0r45))/reference/referencespapers.aspx?referenceid=2534983

[38] Kremer LCM, Leclercq E, van Dalen EC. Cochrane Childhood Cancer Group.About The Cochrane Collaboration (Cochrane Review Groups (CRGs)). 2014. Issue 6. Art. No.: CHILDCA.

[39] Higgins JPT, Altman DG, Gøtzsche PC (2011). The Cochrane Collaboration’s tool for assessing risk of bias in randomised trials. BMJ (Online).

[40] Hamed AA, Hassanen NAA, Mohamed SA (2016). Effect of honey on febrile neutropenia in children with acute lymphoblastic leukemia: A randomized crossover open-labeled study. Complement Ther Med..

[41] Ramezani N, Moafi A, Nadjarzadeh A, Yousefian S, Reisi N, Salehi-Abargouei A (2018). The Effect of Soy Nut Compared to Cowpea Nut on Body Weight, Blood Cells, Inflammatory Markers and Chemotherapy Complications in Children with Acute Lymphoblastic Leukemia: a Randomized Controlled Clinical Trial. Nutr Cancer.

[42] Dagdemir A, Yildirim H, Aliyazicioglu Y, Kanber Y, Albayrak D, Acar S (2004). Does vitamin A prevent high-dose-methotrexate-induced D-xylose malabsorption in children with cancer?. Supportive Care Cancer.

[43] Bordbar M, Shakibazad N, Fattahi M, Haghpanah S, Honar N (2018). Effect of ursodeoxycholic acid and vitamin E in the prevention of liver injury from methotrexate in pediatric leukemia. Turk J Gastroenterol..

[44] Solmaz I, Ozdemir MA, Unal E, Karakukcu M, Abdurrezzak U, Muhtaroglu S. Effect of vitamin K2 and vitamin D3 on bone mineral density in children with acute lymphoblastic leukemia: a prospective cohort study. J Ped Endocrinol Metab. Published online 2021. 10.1515/jpem-2020-0637 10.1515/jpem-2020-0637 33639045

[45] Abu Zaid Z, Shahar S, Jamal AR, Mohd Yusof NA (2012). Fish oil supplementation is beneficial on caloric intake, appetite and mid upper arm muscle circumference in children with leukaemia. Asia Pac J Clin Nutr.

[46] Sands S, Ladas EJ, Kelly KM (2017). Glutamine for the treatment of vincristine-induced neuropathy in children and adolescents with cance. Support Care Cancer.

[47] Li R, Donnella H, Knouse P (2017). A randomized nutrition counseling intervention in pediatric leukemia patients receiving steroids results in reduced caloric intake. Pediatr Blood Cancer.

[48] Widjaja NA, Pratama A, Prihaningtyas R, Irawan R, Ugrasena I (2020). Efficacy oral glutamine to prevent oral mucositis and reduce hospital costs during chemotherapy in children with acute lymphoblastic leukemia. Asian Pac J Cancer Prev..

[49] Zhang F, Wang J, Han Ya of G enriched nutrition therapy in childhood acute lymphoblastic leukemia (2016). Application of Glutamine-enriched nutrition therapy in childhood acute lymphoblastic leukemia. Nutr J..

[50] Rocha KC, Vieira ML, Beltrame RL (2016). Impact of selenium supplementation in neutropenia and immunoglobulin production in childhood cancer patients. J Med Food..

[51] Elbarbary NS, Ismail EAR, Farahat RK, El-Hamamsy M (2016). Omega-3 fatty acids as an adjuvant therapy ameliorates methotrexate-induced hepatotoxicity in children and adolescents with acute lymphoblastic leukemia: a randomized placebo-controlled study. Nutrition..

[52] Consolo LZ, Melnikov P, Cônsolo FZ, Nascimento VA, Pontes VA (2013). Zinc supplementation in children and adolescents with acute leukemia. Eur J Clin Nutr..

[53] Vieira ML, Fonseca FL, Costa LG (2015). Supplementation with selenium can influence nausea, fatigue, physical, renal, and liver function of children and adolescents with cancer. J Med Food..

[54] Orgel E, Mueske NM, Sposto R (2017). A randomized controlled trial testing an adherence-optimized Vitamin D regimen to mitigate bone change in adolescents being treated for acute lymphoblastic leukemia. Leuk Lymphoma..

[55] Hagag AA, Badraia IM, El-Shehaby WA, Mabrouk MM (2020). Protective role of black seed oil in doxorubicin-induced cardiac toxicity in children with acute lymphoblastic leukemia. J Oncol Pharm Pract.

[56] Abdulrhman M, Elbarbary NS, Ahmed Amin D, Saeid Ebrahim R (2012). Honey and a mixture of honey, beeswax, and olive oil-propolis extract in treatment of chemotherapy-induced oral mucositis: a randomized controlled pilot study. Pediatr Hematol Oncol..

[57] Baena-Gómez MA, de la Torre Aguilar MJ, Mesa MD, Llorente-Cantarero FJ, Pérez Navero JL, Gil-Campos M (2013). Effects of parenteral nutrition formulas on plasma lipid profile in children with bone marrow transplantation. Ann Nutr Metab.

[58] Jiménez Jiménez FJ, Ortiz Leyba C, García Garmendia JL, Garnacho Montero J, Rodríguez Fernández JM, Espigado Tocino I (1999). Prospective comparative study of different amino acid and lipid solutions in parenteral nutrition of patients undergoing bone marrow transplantation. Nutr Hosp..

[59] Hagag AA, AbdElaal AM, Elfaragy MS, Hassan SM, Elzamarany EA (2015). Therapeutic value of black seed oil in methotrexate hepatotoxicity in Egyptian children with acute lymphoblastic leukemia. Infect Disord Drug Targets..

[60] Wehner PS, Husby S, Rathe M (2020). Bovine Colostrum Against Chemotherapy-Induced Gastrointestinal Toxicity in Children With Acute Lymphoblastic Leukemia: A Randomized, Double-Blind, Placebo-Controlled Trial. J Parenter Enter Nutr.

[61] Noguera D, Figueroa de Quintero O, Soto de Sanabria I, Nolis C, García JA, Gil ME (2005). Evaluación de la eficacia del soporte nutricional enteral: en niños con leucemia linfocítica aguda de bajo riesgo TT - Evaluation of the enteral nutritional support effectiveness: in children with low risk acute linfocitic. Rev Venez Oncol..

[62] Aquino VM, Jackson GB, Harvey AR (2005). A double-blind randomized placebo-controlled study of oral glutamine in the prevention of mucositis in children undergoing hematopoietic stem cell transplantation: A pediatric blood and marrow transplant consortium study. Bone Marrow Transplant.

[63] Al-Tonbary Y, Al-Haggar M, El-Ashry R, Fouda A, El-Dakroory S, Azzam H (2009). Vitamin e and N-acetylcysteine as antioxidant adjuvant therapy in children with acute lymphoblastic leukemia. Adv Hematol.

[64] Reyna-Figueroa J, Barron-Calvillo E, Garcia-Parra C (2019). Probiotic Supplementation Decreases Chemotherapy-induced Gastrointestinal Side Effects in Patients With Acute Leukemia. J Pediatr Hematol Oncol.

[65] Alves S, Azzalis LA, Gehrke F (2016). Evaluation of biochemical parameters in selenium-supplemented infant patients using non-linear optical method (Z-scan). Tumor Biol..

[66] Wiernikowski JT, Bernhardt MB. Review of nutritional status, body composition, and effects of antineoplastic drug disposition. Pediatr Blood Cancer. 2020;67(S3). 10.1002/pbc.28207 10.1002/pbc.28207 32083372

[67] Tseytlin GJ, Anisimova A V., Godina EZ, et al. Body composition in remission of childhood cancer. In: Journal of Physics: Conference Series. Vol 407. Institute of Physics Publishing; 2012. 10.1088/1742-6596/407/1/012005

[68] Bye A, Sandmael JA, Stene GB (2020). Exercise and nutrition interventions in patients with head and neck cancer during curative treatment: A systematic review and meta-analysis. Nutrients.

[69] Allenby TH, Crenshaw ML, Mathis K (2020). A systematic review of home-based dietary interventions during radiation therapy for cancer. Tech Innov Patient Support Radiat Oncol.

[70] Baguley BJ, Skinner TL, Wright ORL (2019). Nutrition therapy for the management of cancer-related fatigue and quality of life: A systematic review and meta-analysis. Br J Nutr.

[71] Chow R, Bruera E, Chiu L (2016). Enteral and parenteral nutrition in cancer patients: A systematic review and meta-analysis. Ann Palliat Med.

[72] Hamaker ME, Oosterlaan F, van Huis LH, Thielen N, Vondeling A, van den Bos F (2021). Nutritional status and interventions for patients with cancer – A systematic review. J Geriatr Oncol.

[73] Rinninella E, Cintoni M, Raoul P (2020). Effects of nutritional interventions on nutritional status in patients with gastric cancer: A systematic review and meta-analysis of randomized controlled trials. Clin Nutr ESPEN.

[74] Fajardo-Gutiérrez A, Rendón-Macías ME, Mejía-Aranguré JM. Epidemiología Del Cáncer En Niños Mexicanos. Resultados Globales. Rev Med Inst Mex Seguro Soc. 2011;49(Supl 1):S43–S70. 23383475

[75] Viani K, Albuquerque L, Barr RD, Ladas EJ (2020). Nutrition of Children With Cancer in Brazil: A Systematic Review. JCO Global Oncol.

[76] Feng S, Cheng L, Lu H, Shen N (2021). Nutritional Status and Clinical Outcomes in Children with Cancer on Admission to Intensive Care Units. Nutr Cancer.

[77] Ghaffar F, Mehmood N, Khan I, Din ZU, Iqbal Z, Iqbal M (2019). Effects of nutritional intervention and dietary modification on the health status of pediatric acute lymphoblastic leukemia patients. Prog Nutr.

[78] Terwilliger T, Abdul-Hay M (2017). Acute lymphoblastic leukemia: a comprehensive review and 2017 update. Blood Cancer J.

[79] Jansen H, Postma A, Stolk RP, Kamps WA (2009). Acute lymphoblastic leukemia and obesity: Increased energy intake or decreased physical activity?. Support Care Cancer.

[80] Totadri S, Trehan A, Mahajan D, Viani K, Barr R, Ladas EJ. Validation of an algorithmic nutritional approach in children undergoing chemotherapy for cancer. Pediatr Blood Cancer. 2019;66(12). 10.1002/pbc.27980 10.1002/pbc.27980 31464100

[81] Organization PAH (2023). Nutritional Care Guide for Pediatric Cancer. PAHO.

[82] Pedretti L, Massa S, Leardini D, et al. Role of Nutrition in Pediatric Patients with Cancer. Nutrients. 2023;15(3). 10.3390/nu15030710 10.3390/nu15030710 PMC9920596 36771416

[83] Kumari R, Kohli A, Malhotra P, Grover S, Khadwal A (2018). Burden of caregiving and its impact in the patients of acute lymphoblastic leukemia. Ind Psychiatry J.

[84] Tappenden KA, Quatrara B, Parkhurst ML, Malone AM, Fanjiang G, Ziegler TR (2013). Critical role of nutrition in improving quality of care: an interdisciplinary call to action to address adult hospital malnutrition. J Acad Nutr Diet.

[85] Pediatric Quality of Life InventoryTM (PedsQLTM) SCALING AND SCORING OF THE. https://eprovide.mapi-trust.org/

[86] Devilli L, Garonzi C, Balter R (2021). Long-term and quality of survival in patients treated for acute lymphoblastic leukemia during the pediatric age. Hematol Rep.

[87] Guidelines Review Committee, Quality Assurance of Norms and Standards (2014). WHO Handbook for Guideline Development. 2nd ed. (World Health Organization, ed.).

[88] Murad MH, Asi N, Alsawas M, Alahdab F (2016). New evidence pyramid. Evid Based Med.

[89] American Institute of Cancer Research. Diet, Nutrition, Physical Activity and Cancer: A Global Perspective; 2018. http://gco.iarc.fr/today%0Adietandcancerreport.org

[90] Key TJ, Bradbury KE, Perez-Cornago A, Sinha R, Tsilidis KK, Tsugane S (2020). Diet, nutrition, and cancer risk: What do we know and what is the way forward?. The BMJ.

[91] Tripodi SI, Bergami E, Panigari A (2023). The role of nutrition in children with cancer. Tumori.

[92] Chan AW, Tetzlaff JM, Altman DG, et al. SPIRIT 2013 Statement: Defining Standard Protocol Items for Clinical Trials DEVELOPMENT OF THE SPIRIT 2013 STATEMENT. Vol 158.; 2013. www.annals.org

[93] Chan AW, Tetzlaff JM, Gøtzsche PC (2013). SPIRIT 2013 explanation and elaboration: guidance for protocols of clinical trials. BMJ.

[94] Butcher NJ, Monsour A, Mew EJ (2022). Guidelines for Reporting Outcomes in Trial Reports: The CONSORT-Outcomes 2022 Extension. JAMA.

[95] Schulz KF, Altman DG, Moher D, for the CONSORT Group (2010). CONSORT 2010 Statement: updated guidelines for reporting parallel group randomised trials. BMC Med..

