# Supplementary material for: Nutritional interventions in children with acute lymphoblastic leukemia undergoing antineoplastic treatment: a systematic review
Source: BMC Nutr. 2024 Jun 19;10:89. doi: 10.1186/s40795-024-00892-4 (PMC11186292; doi:10.1186/s40795-024-00892-4)
Supplement: Supplementary file 1 — Additional file 1. Search terms and strategy. [file 40795_2024_892_MOESM1_ESM.docx]

**Nutritional interventions in children with acute lymphoblastic leukemia undergoing antineoplastic treatment: a systematic review**

***Supplementary file 1 - ALL search terms and results***

Search terms used when searching for studies – MESH terms, title, abstract and keywords:

1. **Population**

(((child*[Title/Abstract]) OR (adolescent*[Title/Abstract])) OR (childhood[Title/Abstract])) OR (youth*[Title/Abstract]) OR ((((("child"[MeSH Terms]) OR ("child, preschool"[MeSH Terms])) OR ("adolescent"[MeSH Terms])) OR ("minors"[MeSH Terms])) OR ("infant"[MeSH Terms])) OR ("infant, newborn"[MeSH Terms])

1. **Condition/Pathology**

(((((("leukemia"[Title/Abstract]) OR ("leukaemia"[Title/Abstract])) OR ("leucemia"[Title/Abstract])) OR ("leucaemia"[Title/Abstract])) OR (acute lymphoblastic leu*[Title/Abstract])) OR ("blood cancer"[Title/Abstract])) OR (hematologic* malignanc*[Title/Abstract]) OR (("leukemia"[MeSH Terms]) OR ("precursor cell lymphoblastic leukemia lymphoma"[MeSH Terms])) OR ("hematologic diseases"[MeSH Terms])

1. **Study type**

("groups"[Title/Abstract] OR "trial"[Title/Abstract] OR "randomly"[Title/Abstract] OR "drug therapy"[MeSH Subheading] OR "placebo"[Title/Abstract] OR "randomized"[Title/Abstract] OR "controlled clinical trial"[Publication Type] OR "randomized controlled trial"[Publication Type]) NOT (("animals"[MeSH Terms]) NOT ("humans"[MeSH Terms]))

1. **Nutrition**

((((((((((((((((((((diet* therap*[Title/Abstract]) OR (diet* intervention*[Title/Abstract])) OR (diet* treatment*[Title/Abstract])) OR (nutrition* intervention*[Title/Abstract])) OR (nutrition* support*[Title/Abstract])) OR (diet* supplement*[Title/Abstract])) OR (parenteral nutrition[Title/Abstract])) OR (enteral nutrition[Title/Abstract])) OR (supplement*[Title/Abstract])) OR (supplement* feed*[Title/Abstract])) OR (energy intake*[Title/Abstract])) OR (micronutrient*[Title/Abstract])) OR (macronutrient*[Title/Abstract])) OR (nutrient*[Title/Abstract])) OR (vitamin*[Title/Abstract])) OR (mineral*[Title/Abstract])) OR (amino acid*[Title/Abstract])) OR (carbohydrate*[Title/Abstract])) OR (fatty acid*[Title/Abstract])) OR (cholesterol[Title/Abstract])) OR (dietary fiber*[Title/Abstract]) OR (((((((((((((((((("enteral nutrition"[MeSH Terms]) OR ("nutrition therapy"[MeSH Terms])) OR ("parenteral nutrition"[MeSH Terms])) OR ("parenteral nutrition, total"[MeSH Terms])) OR ("dietary supplements"[MeSH Terms])) OR ("energy intake"[MeSH Terms])) OR ("vitamins"[MeSH Terms])) OR ("minerals"[MeSH Terms])) OR ("nutrients"[MeSH Terms])) OR ("amino acids"[MeSH Terms])) OR ("dietary proteins"[MeSH Terms])) OR ("carbohydrates"[MeSH Terms])) OR ("dietary carbohydrates"[MeSH Terms])) OR ("cholesterol"[MeSH Terms])) OR ("cholesterol, dietary"[MeSH Terms])) OR ("dietary fats"[MeSH Terms])) OR ("dietary fiber"[MeSH Terms])) OR ("fatty acids"[MeSH Terms])) OR ("glutamine"[MeSH Terms]) OR (((((((((((("diet therapy"[MeSH Terms]) OR ("nutritional support"[MeSH Terms])) OR ("diet, high fat"[MeSH Terms])) OR ("diet, food, and nutrition"[MeSH Terms])) OR ("diet, fat restricted"[MeSH Terms])) OR ("diet, high protein"[MeSH Terms])) OR ("diet, carbohydrate restricted"[MeSH Terms])) OR ("diet, ketogenic"[MeSH Terms])) OR ("diet, gluten free"[MeSH Terms])) OR ("diet, carbohydrate loading"[MeSH Terms])) OR ("diet"[MeSH Terms])) OR ("diet, protein restricted"[MeSH Terms])) OR ("diet, healthy"[MeSH Terms])

1. **Outcomes**

(((((((((((((((((("body weight"[MeSH Terms]) OR ("body mass index"[MeSH Terms])) OR ("body composition"[MeSH Terms])) OR ("adipose tissue"[MeSH Terms])) OR ("muscle, skeletal"[MeSH Terms])) OR ("deuterium"[MeSH Terms])) OR ("deuterium oxide"[MeSH Terms])) OR ("body water"[MeSH Terms])) OR ("body constitution"[MeSH Terms])) OR ("anthropometry"[MeSH Terms])) OR ("waist circumference"[MeSH Terms])) OR ("waist hip ratio"[MeSH Terms])) OR ("waist height ratio"[MeSH Terms]))) OR ("malnutrition"[MeSH Terms])) OR ("nutritional status"[MeSH Terms])) OR ("protein energy malnutrition"[MeSH Terms]))) OR ("severe acute malnutrition"[MeSH Terms]) OR ((((((((((((((((("body weight"[Title/Abstract]) OR ("body height"[Title/Abstract])) OR ("body mass index"[Title/Abstract])) OR ("body composition"[Title/Abstract])) OR ("body fat"[Title/Abstract])) OR ("fat free mass"[Title/Abstract])) OR ("deuterium"[Title/Abstract])) OR ("deuterium oxide"[Title/Abstract])) OR ("body water"[Title/Abstract])) OR ("waist circumference"[Title/Abstract])) OR ("waist to hip ratio"[Title/Abstract])) OR ("waist hip ratio"[Title/Abstract])) OR ("waist to height"[Title/Abstract])) OR ("waist height ratio"[Title/Abstract])) OR (skinfold*[Title/Abstract]))) OR ("malnutrition"[Title/Abstract])) OR ("sarcopenic obesity"[Title/Abstract])

**Search results for each source – ALL**

| **Database** | **Keyword areas searched** | **Date searched** | **No. refs found** | **No. refs after duplicates removed** | **Potentially meets the inclusion criteria** | **Included** |
| --- | --- | --- | --- | --- | --- | --- |
| **Electronic Databases** |  |  |  |  |  |  |
| Cochrane CENTRAL | 1, 2, 4 | 16/Sep/2021 | 2250 | 2203 | 69 | 20 |
| Embase | 1, 2, 3, 4 | 22/Sep/2021 | 562 | 543 | 7 | 5 |
| LILACS (BVSalud) | 1, 2 | 16/Sep/2021 | 286 | 284 | 3 | 0 |
| MedLine (Ovid) | 1, 2, 3, 4 | 16/Sep/2021 | 619 | 619 | 39 | 0 |
| SciELO | 1, 2 | 16/Sep/2021 | 406 | 388 | 2 | 0 |
| **Total:** | | | 4097 | 4040 | 120 | 25 |
| **Manual searches** |  |  |  |  |  |  |
| Google, Google Scholar, Trial registers, Open Grey | 2, 4 | 16/Sep/2021 | 25 | 0 | 0 | 0 |
| **TOTAL:** | | | 25 | 0 | 0 | 0 |

**Search strategies**

*Cochrane CENTRAL*

ID Search Hits

#1 MeSH descriptor: [Child, Preschool] explode all trees 30102

#2 MeSH descriptor: [Child] explode all trees 57420

#3 MeSH descriptor: [Adolescent] explode all trees 105819

#4 MeSH descriptor: [Minors] explode all trees 10

#5 MeSH descriptor: [Infant] explode all trees 32816

#6 MeSH descriptor: [Infant, Newborn] explode all trees 16362

#7 ("Child"):ti,ab,kw OR ("adolescent"):ti,ab,kw OR ("childhood"):ti,ab,kw OR ("children"):ti,ab,kw OR ("youth"):ti,ab,kw (Word variations have been searched) 249393

#8 #1 or #2 or #3 or #4 or #5 or #6 or #7 261577

#9 ("leukemia"):ti,ab,kw OR ("leukaemia"):ti,ab,kw OR ("leucemia"):ti,ab,kw OR ("leucaemia"):ti,ab,kw (Word variations have been searched) 14436

#10 ("acute lymphoblastic leukemia"):ti,ab,kw OR ("acute lymphoblastic leukaemia"):ti,ab,kw OR ("acute lymphoblastic leucemia"):ti,ab,kw OR ("acute lymphoblastic leucaemia"):ti,ab,kw (Word variations have been searched) 2743

#11 ("blood cancer"):ti,ab,kw OR ("hematologic malignancy"):ti,ab,kw OR ("hematologic malignancies"):ti,ab,kw (Word variations have been searched) 2465

#12 MeSH descriptor: [Precursor Cell Lymphoblastic Leukemia-Lymphoma] explode all trees 1166

#13 MeSH descriptor: [Hematologic Diseases] explode all trees 14843

#14 MeSH descriptor: [Leukemia] explode all trees 4780

#15 #9 or #10 or #11 or #12 or #13 or #14 29010

#16 ("diet therapy"):ti,ab,kw OR ("diet" intervention):ti,ab,kw OR ("diet" treatment):ti,ab,kw OR ("nutrition" intervention):ti,ab,kw OR ("nutrition" support):ti,ab,kw (Word variations have been searched) 63413

#17 ("diet" "supplement"):ti,ab,kw OR ("parenteral nutrition"):ti,ab,kw OR ("enteral nutrition"):ti,ab,kw OR ("supplement" "feed"):ti,ab,kw (Word variations have been searched) 24698

#18 ("energy intake"):ti,ab,kw OR ("micronutrient"):ti,ab,kw OR ("macronutrient"):ti,ab,kw OR ("vitamin"):ti,ab,kw OR ("mineral"):ti,ab,kw (Word variations have been searched) 54253

#19 ("amino acid"):ti,ab,kw OR ("carbohydrate"):ti,ab,kw OR ("fatty acid"):ti,ab,kw OR ("cholesterol"):ti,ab,kw OR (dietary fiber):ti,ab,kw (Word variations have been searched) 73518

#20 MeSH descriptor: [Diet Therapy] explode all trees 6071

#21 MeSH descriptor: [Nutritional Support] explode all trees 3442

#22 MeSH descriptor: [Diet, High-Fat] explode all trees 267

#23 MeSH descriptor: [Diet] explode all trees 18928

#24 MeSH descriptor: [Diet, Food, and Nutrition] explode all trees 55221

#25 MeSH descriptor: [Diet, Fat-Restricted] explode all trees 981

#26 MeSH descriptor: [Diet, High-Protein] explode all trees 77

#27 MeSH descriptor: [Diet, Carbohydrate-Restricted] explode all trees 453

#28 MeSH descriptor: [Diet, Ketogenic] explode all trees 75

#29 MeSH descriptor: [Diet, Gluten-Free] explode all trees 98

#30 MeSH descriptor: [Nutrition Therapy] explode all trees 9636

#31 MeSH descriptor: [Diet, Protein-Restricted] explode all trees 221

#32 MeSH descriptor: [Diet, Healthy] explode all trees 522

#33 MeSH descriptor: [Dietary Supplements] explode all trees 13042

#34 MeSH descriptor: [Parenteral Nutrition] explode all trees 1674

#35 MeSH descriptor: [Parenteral Nutrition, Total] explode all trees 766

#36 MeSH descriptor: [Enteral Nutrition] explode all trees 1890

#37 MeSH descriptor: [Energy Intake] explode all trees 5498

#38 MeSH descriptor: [Vitamins] explode all trees 4833

#39 MeSH descriptor: [Minerals] explode all trees 3898

#40 MeSH descriptor: [Nutrients] explode all trees 5332

#41 MeSH descriptor: [Amino Acids] explode all trees 21896

#42 MeSH descriptor: [Dietary Proteins] explode all trees 4205

#43 MeSH descriptor: [Carbohydrates] explode all trees 60370

#44 MeSH descriptor: [Dietary Carbohydrates] explode all trees 6463

#45 MeSH descriptor: [Cholesterol] explode all trees 10311

#46 MeSH descriptor: [Cholesterol, Dietary] explode all trees 283

#47 MeSH descriptor: [Dietary Fats] explode all trees 7624

#48 MeSH descriptor: [Dietary Fiber] explode all trees 2052

#49 MeSH descriptor: [Fatty Acids] explode all trees 22718

#50 MeSH descriptor: [Dietary Supplements] explode all trees 13042

#51 MeSH descriptor: [Micronutrients] explode all trees 5901

#52 MeSH descriptor: [Glutamine] explode all trees 661

#53 (glutamine):ti,ab,kw (Word variations have been searched) 2079

#54 #16 or #17 or #18 or #19 or #20 or #21 or #22 or #23 or #24 or #25 or #26 or #27 or #28 or #29 or #30 or #31 or #32 or #33 or #34 or #35 or #36 or #37 or #38 or #39 or #40 or #41 or #42 or #43 or #44 or #45 or #46 or #47 or #48 or #49 or #50 or #51 or #52 or #53 247411

#55 #8 AND #15 AND #54 2250

*MedLine*

1 exp Child/ 1974996

2 exp Child, Preschool/ 944939

3 exp Adolescent/ 2097307

4 exp Minors/ 2645

5 exp Infant/ 1171670

6 exp Infant, newborn/ 626342

7 "child*".ab,kw,ti. 1482027

8 "adolescent*".ab,kw,ti. 277469

9 childhood.ab,kw,ti. 251586

10 "youth*".ab,kw,ti. 87255

11 1 or 2 or 3 or 4 or 5 or 6 or 7 or 8 or 9 or 10 4096865

12 Leukemia/ or Precursor Cell Lymphoblastic Leukemia-Lymphoma/ 83360

13 Hematologic Diseases/ 18549

14 (leukemia or leukaemia or leucemia or leucaemia).ab,kw,ti. 254995

15 "acute lymphoblastic leu*".ab,kw,ti. 33023

16 "blood cancer*".ab,kw,ti. 1046

17 "hematologic* malignanc*".ab,kw,ti. 22783

18 12 or 13 or 14 or 15 or 16 or 17 312823

19 randomi?ed controlled trial.pt. 533373

20 controlled clinical trial.pt. 94207

21 randomi?ed.ab. 626055

22 placebo.ab. 218865

23 drug therapy.fs. 2329391

24 randomly.ab. 359553

25 trial.ab. 555895

26 groups.ab. 2207212

27 19 or 20 or 21 or 22 or 23 or 24 or 25 or 26 5045577

28 exp animals/ not humans.sh. 4841291

29 27 not 28 4389315

30 Diet Therapy/ 10746

31 Nutritional Support/ 6589

32 Diet, High-Fat/ or "Diet, Food, and Nutrition"/ or Diet, Fat-Restricted/ or Diet, High-Protein/ or Diet, Carbohydrate-Restricted/ or Diet, High-Protein Low-Carbohydrate/ or Diet, Ketogenic/ or Diet, Gluten-Free/ or Diet, Carbohydrate Loading/ or Diet/ or Diet, Protein-Restricted/ or Diet, Healthy/ 199152

33 Enteral Nutrition/ or Nutrition Therapy/ or Parenteral Nutrition/ or Parenteral Nutrition, Total/ 42040

34 Dietary Supplements/ 62671

35 Energy Intake/ 42003

36 Vitamins/ 32258

37 Minerals/ 24548

38 Nutrients/ 3565

39 Amino Acids/ 139303

40 Dietary Proteins/ 38226

41 Dietary Carbohydrates/ or Carbohydrates/ 59157

42 Cholesterol/ or Cholesterol, Dietary/ 126469

43 Dietary Fats/ 48532

44 Dietary Fiber/ 17920

45 Fatty Acids/ 88408

46 Glutamine/ 17835

47 "diet* therap*".ab,kw,ti. 5685

48 "diet* intervention*".ab,kw,ti. 9764

49 "diet* treatment*".ab,kw,ti. 11367

50 "nutrition* intervention*".ab,kw,ti. 7863

51 "nutrition* support*".ab,kw,ti. 11563

52 "diet* supplement*".ab,kw,ti. 31021

53 parenteral nutrition.ab,kw,ti. 20993

54 enteral nutrition.ab,kw,ti. 9505

55 "supplement* feed*".ab,kw,ti. 1935

56 "energy intake*".ab,kw,ti. 23021

57 "micronutrient*".ab,kw,ti. 17465

58 "macronutrient*".ab,kw,ti. 10528

59 "vitamin*".ab,kw,ti. 231763

60 "mineral*".ab,kw,ti. 185232

61 "amino acid*".ab,kw,ti. 496919

62 "carbohydrate*".ab,kw,ti. 145977

63 "fatty acid*".ab,kw,ti. 230962

64 cholesterol.ab,kw,ti. 251142

65 "dietary fiber*".ab,kw,ti. 8183

66 "Glutamine".ab,kw,ti. 38454

67 30 or 31 or 32 or 33 or 34 or 35 or 36 or 37 or 38 or 39 or 40 or 41 or 42 or 43 or 44 or 45 or 46 or 47 or 48 or 49 or 50 or 51 or 52 or 53 or 54 or 55 or 56 or 57 or 58 or 59 or 60 or 61 or 62 or 63 or 64 or 65 or 66 1886987

68 11 and 18 and 29 and 67 619

*LILACS*

(child* OR adolescent* OR minor* OR childhood OR youth*)

AND

(leukemia OR leukaemia OR leucemia OR leucaemia OR “acute lymphoblastic leukemia” OR “acute lymphoblastic leukaemia” OR “acute lymphoblastic leucemia” OR “acute lymphoblastic leucaemia” OR “blood cancer”)

**286**

*SciELO*

(child* OR adolescent* OR minor* OR childhood OR youth*)

AND

(leukemia OR leukaemia OR leucemia OR leucaemia )

**406**

*Embase*

Embase Classic+Embase <1947 to 2021 September 21>

1 diet, high-fat/ 51239

2 exp Child/ 3202365

3 exp Child, Preschool/ 654009

4 exp Adolescent/ 1758352

5 exp Minors/ 753

6 exp Infant/ 1218417

7 exp Infant, newborn/ 645882

8 "child*".ab,kw,ti. 2064049

9 "adolescent*".ab,kw,ti. 378989

10 childhood.ab,kw,ti. 357044

11 "youth*".ab,kw,ti. 107593

12 2 or 3 or 4 or 5 or 6 or 7 or 8 or 9 or 10 or 11 4594002

13 Leukemia/ or Precursor Cell Lymphoblastic Leukemia-Lymphoma/ 130400

14 Hematologic Disease/ 29682

15 (leukemia or leukaemia or leucemia or leucaemia).ab,kw,ti. 375463

16 "acute lymphoblastic leu*".ab,kw,ti. 51535

17 "blood cancer*".ab,kw,ti. 2800

18 "hematologic* malignanc*".ab,kw,ti. 42849

19 13 or 14 or 15 or 16 or 17 or 18 470472

20 Diet Therapy/ 62125

21 Nutritional Support/ 20659

22 lipid diet/ or "Diet, Food, and Nutrition"/ or Diet, Fat-Restricted/ or protein diet/ or Diet, Carbohydrate-Restricted/ or high-protein low-carbohydrate diet/ or ketogenic diet/ or Gluten-Free diet/ or Carbohydrate Loading diet/ or Diet/ or protein restriction/ or healthy diet/ 470815

23 enteric feeding/ or diet therapy/ or parenteral nutrition/ or total parenteral nutrition/ 133401

24 Dietary Supplement/ 16920

25 caloric intake/ 68104

26 Vitamin/ 63133

27 Mineral/ 50452

28 Nutrient/ 47497

29 Amino Acid/ 211168

30 protein intake/ 46442

31 carbohydrate intake/ or Carbohydrate/ 102998

32 Cholesterol/ or Cholesterol intake/ 234660

33 fat intake/ 51964

34 Dietary Fiber/ 23746

35 Fatty Acid/ 141297

36 Glutamine/ 47108

37 "diet* therap*".ab,kw,ti. 6449

38 "diet* intervention*".ab,kw,ti. 14299

39 "diet* treatment*".ab,kw,ti. 14209

40 "nutrition* intervention*".ab,kw,ti. 11555

41 "nutrition* support*".ab,kw,ti. 17693

42 "diet* supplement*".ab,kw,ti. 40211

43 parenteral nutrition.ab,kw,ti. 31799

44 enteral nutrition.ab,kw,ti. 16973

45 "supplement* feed*".ab,kw,ti. 2052

46 "energy intake*".ab,kw,ti. 30542

47 "micronutrient*".ab,kw,ti. 23525

48 "macronutrient*".ab,kw,ti. 14097

49 "vitamin*".ab,kw,ti. 326628

50 "mineral*".ab,kw,ti. 246890

51 "amino acid*".ab,kw,ti. 570545

52 "carbohydrate*".ab,kw,ti. 186984

53 "fatty acid*".ab,kw,ti. 290574

54 cholesterol.ab,kw,ti. 357688

55 "dietary fiber*".ab,kw,ti. 10439

56 "Glutamine".ab,kw,ti. 48411

57 20 or 21 or 22 or 23 or 24 or 25 or 26 or 27 or 28 or 29 or 30 or 31 or 32 or 33 or 34 or 35 or 36 or 37 or 38 or 39 or 40 or 41 or 42 or 43 or 44 or 45 or 46 or 47 or 48 or 49 or 50 or 51 or 52 or 53 or 54 or 55 or 56 2623879

58 12 and 19 and 57 2866

59 Randomized controlled trial/ 679563

60 Controlled clinical trial/ 464412

61 random$.ti,ab. 1719932

62 randomization/ 92122

63 intermethod comparison/ 275419

64 placebo.ti,ab. 334591

65 (compare or compared or comparison).ti. 577987

66 ((evaluated or evaluate or evaluating or assessed or assess) and (compare or compared or comparing or comparison)).ab. 2379182

67 (open adj label).ti,ab. 91075

68 ((double or single or doubly or singly) adj (blind or blinded or blindly)).ti,ab. 254097

69 double blind procedure/ 190514

70 parallel group$1.ti,ab. 28152

71 (crossover or cross over).ti,ab. 114095

72 ((assign$ or match or matched or allocation) adj5 (alternate or group$1 or intervention$1 or patient$1 or subject$1 or participant$1)).ti,ab. 365619

73 (assigned or allocated).ti,ab. 431045

74 (controlled adj7 (study or design or trial)).ti,ab. 392193

75 (volunteer or volunteers).ti,ab. 266973

76 human experiment/ 555788

77 trial.ti. 347646

78 or/59-77 5601264

79 ..nlpx "query=(random* adj sampl* adj7 (cross section* or questionnaire*1 or survey* or database*1)).ti,ab. not (comparative study/ or controlled study/ or randomised controlled.ti,ab. or randomized controlled.ti,ab. or randomly assigned.ti,ab.) ","desiredResults=10000","minHitsDivisor=7","permitHyponyms=NO","lowestVocabularySearchLevel=none","phrasesBroken=NO","speedWanted=Fastest","comment=No Related Terms","elimEnable=NO","constraintMinTerms=1" 146

80 ..nlpx "query=Cross-sectional study/ not (randomized controlled trial/ or controlled clinical study/ or controlled study/ or randomized controlled.ti,ab. or randomised controlled.ti,ab. orcontrol group$1.ti,ab.)","desiredResults=10000","minHitsDivisor=7","permitHyponyms=NO","lowestVocabularySearchLevel=none","phrasesBroken=NO","speedWanted=Fastest","comment=No Related Terms","elimEnable=NO","constraintMinTerms=1" 10420

81 (((case adj control$) and random$) not randomi#ed controlled).ti,ab. 18940

82 (Systematic review not (trial or study)).ti. 186830

83 (nonrandom$ not random$).ti,ab. 17386

84 Random field$.ti,ab. 2585

85 (random cluster adj3 sampl$).ti,ab. 1381

86 (review.ab. and review.pt.) not trial.ti. 927712

87 ..nlpx "query=we searched.ab. and (review.ti. or review.pt.)","desiredResults=10000","minHitsDivisor=7","permitHyponyms=NO","lowestVocabularySearchLevel=none","phrasesBroken=NO","speedWanted=Fastest","comment=No Related Terms","elimEnable=NO","constraintMinTerms=1" 0

88 update review.ab. 117

89 (databases adj4 searched).ab. 45375

90 (rat or rats or mouse or mice or swine or porcine or murine or sheep or lambs or pigs or piglets or rabbit or rabbits or cat or cats or dog or dogs or cattle or bovine or monkey or monkeys or trout or marmoset$1).ti. and animal experiment/ 1122880

91 ..nlpx "query=Animal experiment/ not (human experiment/ or human/)","desiredResults=10000","minHitsDivisor=7","permitHyponyms=NO","lowestVocabularySearchLevel=none","phrasesBroken=NO","speedWanted=Fastest","comment=No Related Terms","elimEnable=NO","constraintMinTerms=1" 63452

92 or/79-91 2266312

93 78 not 92 5194934

94 12 and 19 and 57 and 93 562
