# Supplementary material for: Nutritional interventions in children with acute lymphoblastic leukemia undergoing antineoplastic treatment: a systematic review
Source: BMC Nutr. 2024 Jun 19;10:89. doi: 10.1186/s40795-024-00892-4 (PMC11186292; doi:10.1186/s40795-024-00892-4)
Supplement: Supplementary file 4 — Additional file 4. (Table S1). [file 40795_2024_892_MOESM4_ESM.docx]

Table S1. Characteristics of included studies for the Systematic Review (PICOS criteria: participants, interventions, comparisons outcomes, and study design).

| **Author, year (reference)** | **Country and years of study** | **Participants: number, age, % males** | **Intervention** | **Evaluated outcomes** |  |
| --- | --- | --- | --- | --- | --- |
| ***Compound/Food***  **Black seed oil** | | | | | |
| Hagag, 2015 ^59^ | Egypt 2010-2014 | n=40 (100% ALL) 4-13 years 70% males | Intervention: “Black seed oil in the form of soft gelatin Baraka capsule 450 mg [Pharco Pharmaceuticals] in dose of 80 mg/kg/day on three divided doses for one week after each HDMTX in all phases of chemotherapy” Control: “patients under HDMTX therapy, delayed leukovorin rescue and placebo for one week after giving HDMTX therapy without black seed oil” | ALT (UI/L) AST (UI/L) ALP (UI/L) Total serum protein (g/dL) Serum albumin (mg/dL) Total bilirubin (mg/dL) |  |
| Hagag, 2020 ^55^ | Egypt 2016-2018 | n=40 (100% ALL) 2-16 years 63% males | Intervention: “Black seed oil 80mg/kg/dose divided into three doses starting at the same moment of beginning of DOX infusion therapy and continued for one week after each DOX dose (Black seed oil is available in capsule form named Baraka capsule containing 100 or 450mg black seed oil in a box containing 24 capsules)” Control: no black seed oil | Creatinine (mg/dL) Urea (mg/dL) BUN (mg/dL) |  |
| **Glutamine** | | | | | |
| Aquino, 2005 ^62^ | USA 1998-2002 | n=120 (52% ALL) ˂21 years 61% males | Intervention: glutamine Control: glycine “Both groups were administered 2 g/m^2^/dose (maximum dose 4 g) in a solution of 500 mg/mL twice daily. The drug was dissolved in water or other liquid at the local institution. The solution had the consistency of a thickened liquid” | Episodes of patients who developed bacteremia Days of intravenous narcotic use Days of TOTAL PN use Hospital days |  |
| Han, 2016 ^49^ | China 2013-2014 | n=48 (100% ALL) 1-11 years 58% males | Intervention: “glutamine-enriched (0.4 g/kg) nutritional therapy every day during the full course of chemotherapy” Control: as same as the treatment group except without glutamine | Weight (Kg) Triceps skinfold (mm) Serum albumin (g/L) Serum pre-albumin (mg/L) Retinol binding protein (mg/L) Presence of edema |  |
| Sands, 2017 ^46^ | USA Not reported | n=49 (59% ALL)  5-21 years 47% males | Intervention: glutamine  Control: placebo (L-glycine)  “Both groups were administered at a dose of 6 g/m^2^ twice daily (up to a maximum of 10 g/dose) for 21 days” | Presence of sensory neuropathy Presence of motor neuropathy Side effects |  |
| Widjaja, 2020 ^48^ | Indonesia Not reported | n=48 (100% ALL) 1-18 years 65% males | Intervention: glutamine Control: placebo  “Both groups were administered at a dose of 400 mg/kg/day orally, 24 hours before HDMTX for 14 days” | Occurrence of oral mucositis Hospital days |  |
| **Honey** | | | | | |
| Abdulrhman, 2012 ^56^ | Egypt 2010-2011 | n=90 (100% ALL) 2-18 years 63% males | “Intervention 1 (included in the SR): received 0.5 g honey/kg (maximum 15 g) applied topically to the affected oral mucosa 3 times daily until healing or for 10 days, whichever comes first Control: served as controls and received benzocaine 7.5% gel applied topically to the affected oral mucosa 3 times daily” | Recovery time |  |
| Abdulrhman, 2016 ^40^ | Egypt 2011-2013 | n=40 (100% ALL) 2.5-10 years 50% males | Intervention: “2 mL (2.5 g) honey/kg/dose twice weekly for a 12-week period” Control: subjects did not receive honey as a control | Hemoglobin (g/dL) Episodes of febrile neutropenia Number (%) patients who developed febrile neutropenia Hospital days |  |
| **Probiotics** | | | | | |
| Reyna-Figueroa, 2019 ^64^ | Mexico Not reported | n=60 (100% ALL) ˂17 years 63% males | Intervention: “probiotic Lactobacillus *rhamnosus* GG. A concentration of 5×109 CFU per sachet with maltodextrin as excipient was administered at a standard dose (1 sachet) twice daily, by mouth; the probiotic was removed upon completion of a 7-day probiotic course (according to the recommendation of the product), completion of chemotherapy, or neutropenia onset (as long as they do not complete <5 days)” Control: not given probiotics | Constipation Abdominal distention Meteorism  Diarrhea Vomiting  Dyspepsia Nausea |  |
| **Soy nut powder** | | | | | |
| Ramezani, 2018 ^41^ | Iran 2016-2017 | n=56 (100% ALL) 3-12 years 64% males | Intervention: soy nut  Control: cowpea  “Both groups were administered (powders) at a dose of 30g sachets, and they were recommended to consume one sachet along with their food every day for 12 weeks” | Weight (Kg) BMI (Kg/m^2^) Hemoglobin (g/dL) Waist circumference (cm) Pain Fatigue Nausea Depression Anxiety Drowsiness Appetite Well being Dyspnea |  |
| **ω-3** | | | | | |
| Abu Zaid, 2012 ^45^ | Malaysia 2005 | n=51 (100% ALL) 4-12 years 62.7 % males | Intervention: “consumed one capsule of fish oil per day (1200 mg containing 360 mg EPA and 240 mg docosahexaenoic acid)” Control: received a placebo without fish oil | Weight (Kg) MUAC (cm) |  |
| Baena-Gómez, 2013 ^57^ | Spain Not reported | n=14 (57% ALL) 6-14 years 50% males | Intervention: “ω3 LCPUFAS formula: 200 mg/ml (20%) of triglycerides. 10 g of MCT, 8 g of soybean oil and 2 g of triglycerides with ω3 fatty acids per 100 ml. Essential FA: linoleic FA (ω6) 25.72% (5.14 g/100 ml), alpha-linolenic FA (ω3) 3.41% (0.68 g/100 ml), oleic FA 13.44% (2.69 g/100 ml), EPA 3.69% (0.74 g/100 ml), docosahexaenoic FA (ω3) 2.53% (0.51 g/100 ml), ratio ω3/ω6 1: 2.7” Control: “20 g of purified soybean oil per 100 ml. The FA composition was linoleic acid (18: 2 ω6) 52%, alpha-linolenic FA (18: 3 ω3) 8%, oleic FA (18: 1 ω9) 22%, palmitic FA (16: 0) 13%, stearic FA (18: 0) 4%, other FA 1%” | Cholesterol (mg/dL) Triglycerides (mg/dL) HDL (mg/dL) LDL (mg/dL) |  |
| Elbarbary, 2016 ^51^ | Egypt Not reported | n=65 (100% ALL) 4-16 years 55% males | Intervention: “oral ω-3 capsules (Super-omega, Safe Pharma, Alexandria, Egypt), in addition to HDMTX. The ω-3 capsules contained fish oil 1000 mg (180 mg EPA and 120 mg DHA) for 6 months” Control: “included standard-risk patients with ALL who were in maintenance phase (day 0) and receiving oral HDMTX (20 mg/m2) weekly” | Total bilirubin (mg/dL) ALP (UI/L) ALT (UI/L) AST (UI/L) |  |
| **Whey protein hydrolysate** | | | | | |
| Rathe, 2019 ^60^ | Denmark 2013-2016 | n=62 (100% ALL) 1-18 years 52% males | Intervention: “colostrum dose as 1 sachet containing 7.5 g for children (0–15 kg), 2 sachets (15.1–30 kg), 3 sachets (30.1–45 kg), or 4 sachets (>45 kg)” Control: as same as the treatment group except without colostrum “The supplements were to be administered either by mouth or by nasogastric tube and were given as a single dose or divided into 2 or 3 daily doses, depending on the preferences of the child” | Patients with febrile neutropenia, N (%) Days with febrile neutropenia among patients experiencing febrile neutropenia, median (IQR) Oral mucositis Abdominal pain Diarrhea |  |
| ***Micronutrient***  **Selenium** | | | | | |
| Vieira, 2014 ^53^ | Brazil 2010-2012 | n=19 (68% ALL) 0-18 years 63% males | Intervention: received daily supplementation of Se according to the age group presented Control: received placebo capsules In both cases, administration lasted 30 days | Fatigue Nausea Appetite loss Physical function |  |
| Rocha, 2016 ^50^ | Brazil Not reported | n=36 (50% ALL) 0-18 years 64% males | Intervention: “daily Se glycinate (Se molecule was chemically bound to glycine to ameliorate the intestinal absorption) Control: received glycine starch capsules (placebo).  The capsules used in both groups were identical in size and glycine dose amount, with no difference in the storage containers.”  “Dosage administration of Se corresponded to the daily value intake recommended by the DRI for children within the studied age group. For each DRI, an 80% overload was added, and the following doses were established: 27, 36, 54, 72, and 100 ug of Se glycinenate” | Hemoglobin (g/dL) Febrile neutropenia cases |  |
| **Vitamin A** | | | | | |
| Dagdemir, 2004 ^42^ | Turkey 2000-2004 | n=35 (69% ALL) 2.5-16 years 57% males | Intervention: received a dose of 180,000 IU vitamin A once 24 h before HDMTX Control: received only HDMTX | Gastrointestinal toxicity grade Hematological toxicity grade Skin toxicity grade Systemic toxicity grade |  |
| **Vitamin D** | | | | | |
| Orgel, 2017 ^54^ | USA 2011-2014 | n=49 (100% ALL) 10-21 years 61% males | Intervention 1 (included in the SR): “received oral high dose Vitamin D3 100,000IU (10,000IU/1ml, Douglas Laboratories, Pittsburgh, PA) administered in the clinic setting at the start of each of the three included chemotherapy phases (~2-month intervals)”  Control: “all subjects enrolled but not eligible for the open-label randomization (i.e., those Vitamin D sufficient at end of Induction, failure to meet organ function criteria, or documented non-adherence to the initial formulation) were followed as an internal natural-history group to further explore potential associations” | Corrected Ca (mg/dL) Phosphorus (mg/dL) Vitamin D (ng/mL) |  |
| Solmaz, 2021 ^44^ | Turkey 2011-2012 | n=29 (100% ALL) 1-17 years 59% males | Intervention: “was given vitamin K2 (menaquinone-7 100 mcg/day) and vitamin D3 (calcitriol 10 mcg/day) orally as a single morning dose from day 1 of treatment” Control: received no supplement | Ca (mg/dL) P (mg/dL) Mg (mg/dL) ALP (u/L) |  |
| **Vitamin E** | | | | | |
| Al-Tonbary, 2009 ^63^ | Egypt 2006-2007 | n=40 (100% ALL) 2-17 years 45% males | Intervention: “supplemented with vitamin E in a dose of 400 IU/day orally and N-acetylcysteine (NAC) in a dose of 600 mg/day orally in addition to chemotherapy from day one of treatment till the end of intensification phase” Control: received chemotherapy and radiotherapy without any supplementation | Hematological complications N (%) |  |
| Bordbar, 2018 ^43^ | Iran 2014-2015 | n=28 (100% ALL) 2-18 years 66.7% males | Intervention 1 (included in the SR): “treated with vitamin E (E-Zavit, 400 milligrams Capsule) daily along with their routine chemotherapy drugs”  Control: not treated with any drug except their routine chemotherapy agents | Hemoglobin (g/dL) Serum protein (g/dL) Serum albumin (g/L) |  |
| **Zinc** | | | | | |
| Consolo, 2013 ^52^ | Brazil 2010-2012 | n=38 (82% ALL) 1-18 years 53% males | Intervention: “syrup containing Zn in the form of a chelate solution (10 mg/ml) with a dose of 2 mg/kg/day of Zn (maximum 60 mg/day) in two divided doses” Control: given orally placebo syrup containing no Zn | Weight gain (Kg) Presence of oral Mucositis |  |
| ***Nutritional support***  **Enteral Nutrition** | | | | | |
| Noguera, 2005 ^61^ | Venezuela 2010 | n=16 (100% ALL) ˃3 years 62% males | Intervention: “patients whose individual nutritional requirements were covered with the usual diet and polymeric enteral formula (Fortisón® Nutricia Line. Röemmers Laboratories). This formula represented for each case, 30% of the individual caloric requirement” Control: “patients whose individual nutritional requirements were covered exclusively with the usual diet, according to dietary calculations” | Weight (kg) MUAC (cm) BMI (Kg/m^2^) |  |
| **Individualized nutritional counseling** | | | | | |
| Li, 2016 ^47^ | USA Not reported | n=22 (100% ALL) 7-18 years 64% males | Intervention: “counseling intervention consisted of a mandatory baseline visit with the research dietitian, regardless of nutritional needs, and a total of 12 monthly follow-up nutrition counseling sessions” Control: “participants were provided with standard care, and nutrition handouts were available upon request. Standard care was inclusive of nutrition consultation requested by the patient, referral by a physician, or a nutrition screening completed by the nursing staff when the patient showed nutritional risk(s)” | BMI (Kg/m^2^) Waist circumference (cm) |  |
| **Parenteral nutrition** | | | | | |
| Jiménez, 1999 ^74^ | Spain Not reported | n=62 (50% ALL) Not reported 63% males | Intervention 1: (included in the SR) “patients with TOTAL PN with a solution containing 45% AAR plus medium chain triglycerides (MCT/LCT 20%. Lipofundin. Braun)”  Control: “TOTAL PN was administered with standard AA solution (22.5% AA). Freamine (Pharmacia-Upjhon) plus 20% LCT” | Number of infections Serum prealbumin (g/L) Serum albumin (g/dL) Transferrin (mg/dL) Retinol binding protein (mg/L) Serum cholesterol (mg/dL) HDL (mg/dL) LDL (mg/dL) Triglycerides (mg/dL) |  |

ALL, acute lymphoblastic leukemia; ALP, alkaline phosphatase; ALT, alanine aminotransferase; AST, aspartate aminotransferase; BMI, body mass index; BUN, blood urea nitrogen; Ca, calcium; DHA, docosahexaenoic acid; DRI, dietary reference intakes; DOX, doxorubicin, EPA, eicosapentaenoic acid; HDL, high-density lipoprotein; HDMTX, high-dose methotrexate; LCPUFAS, long-chain polyunsaturated fatty acids; LDL, low-density lipoprotein; LCT, long-chain triglycerides; MCT, mid-chain triglycerides; Mg, magnesium; MUAC, mid-upper arm circumference; NAC, N-acetylcysteine; NS, no significative difference; RR, relative risk; P, phosphorus.
