# Supplementary figures and images for: Nutritional interventions in children with acute lymphoblastic leukemia undergoing antineoplastic treatment: a systematic review
Source: BMC Nutr. 2024 Jun 19;10:89. doi: 10.1186/s40795-024-00892-4 (PMC11186292; doi:10.1186/s40795-024-00892-4)

## Slide 1
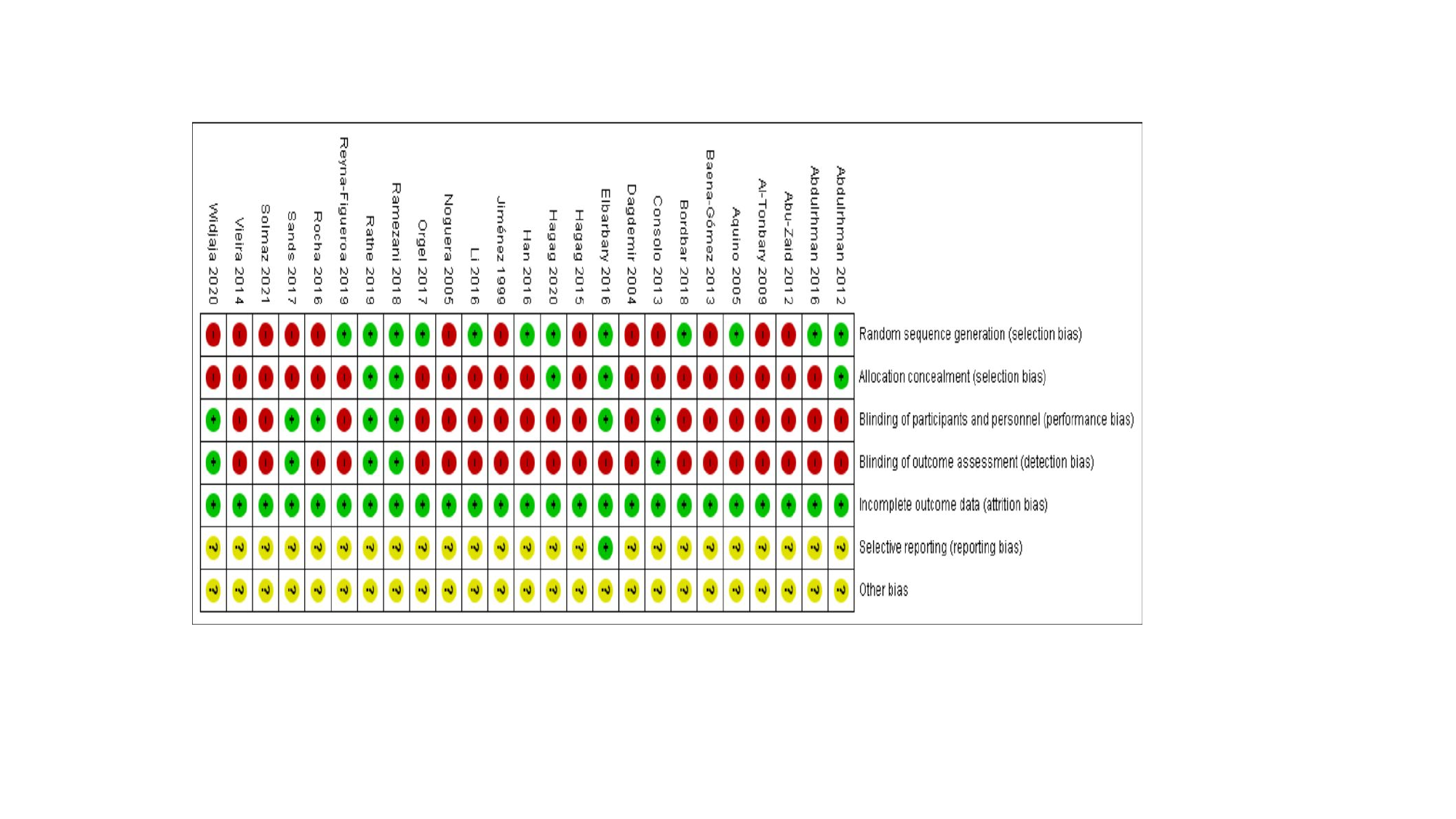

## Slide 2
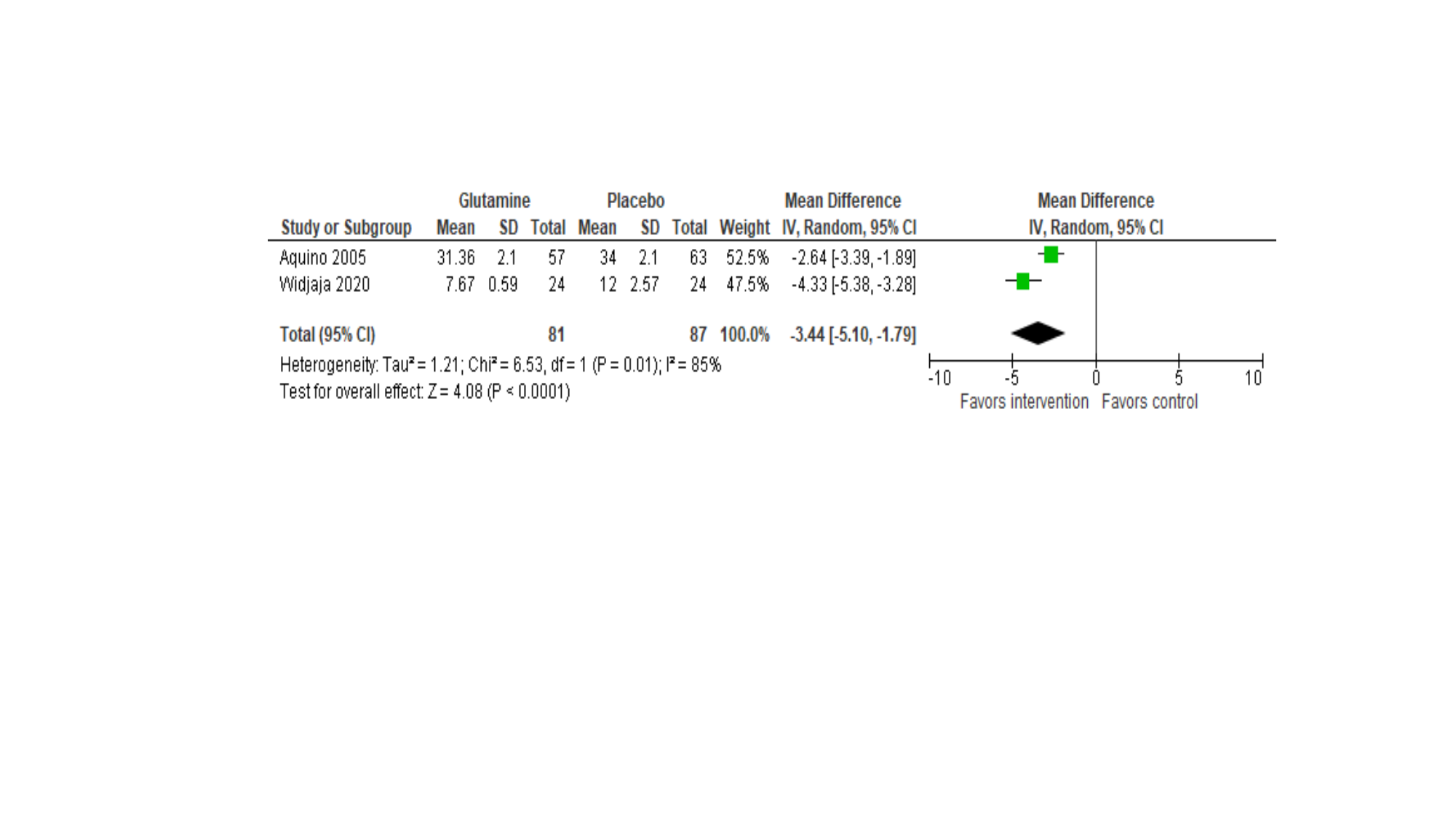

## Slide 3
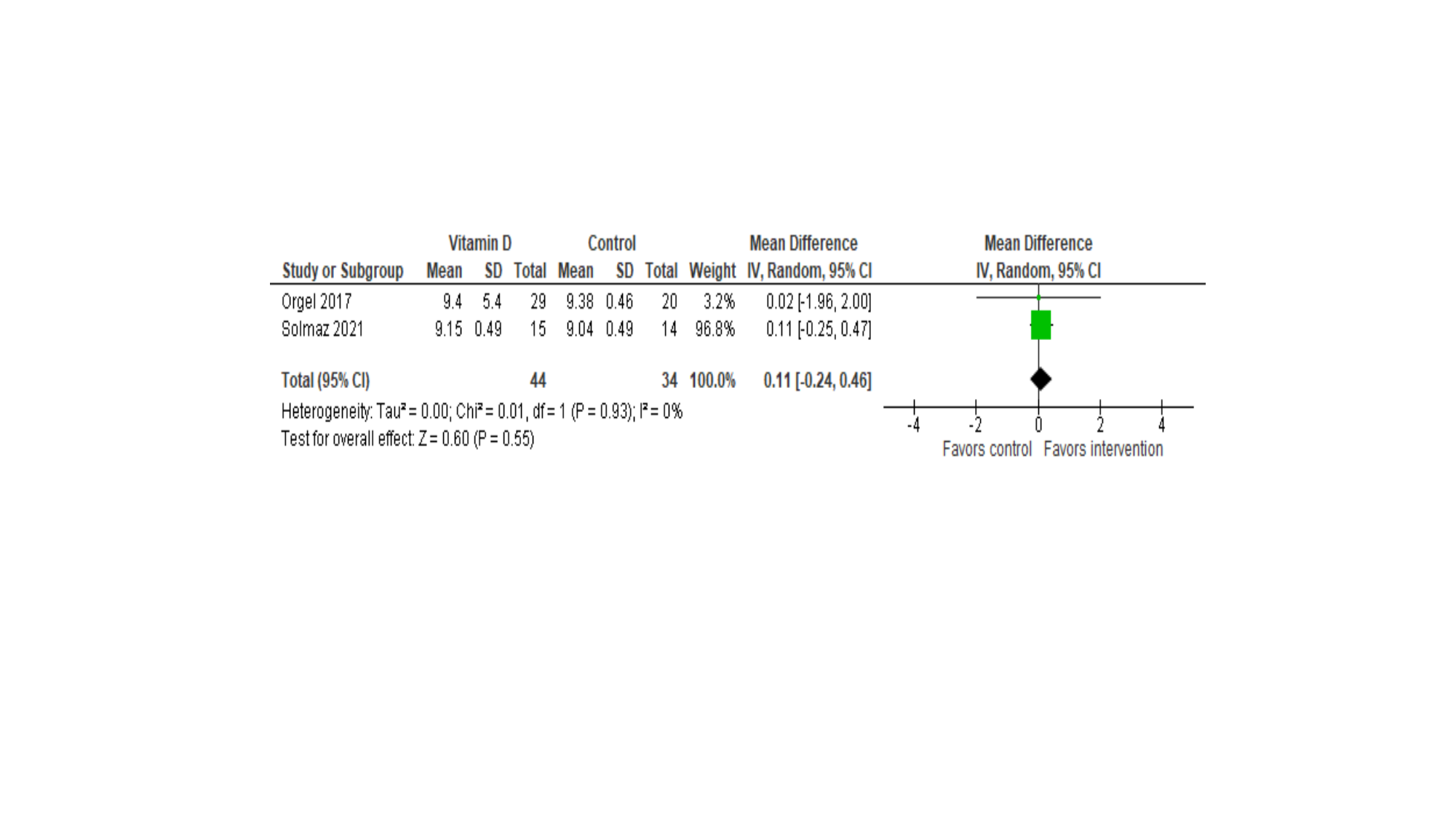

## Slide 4
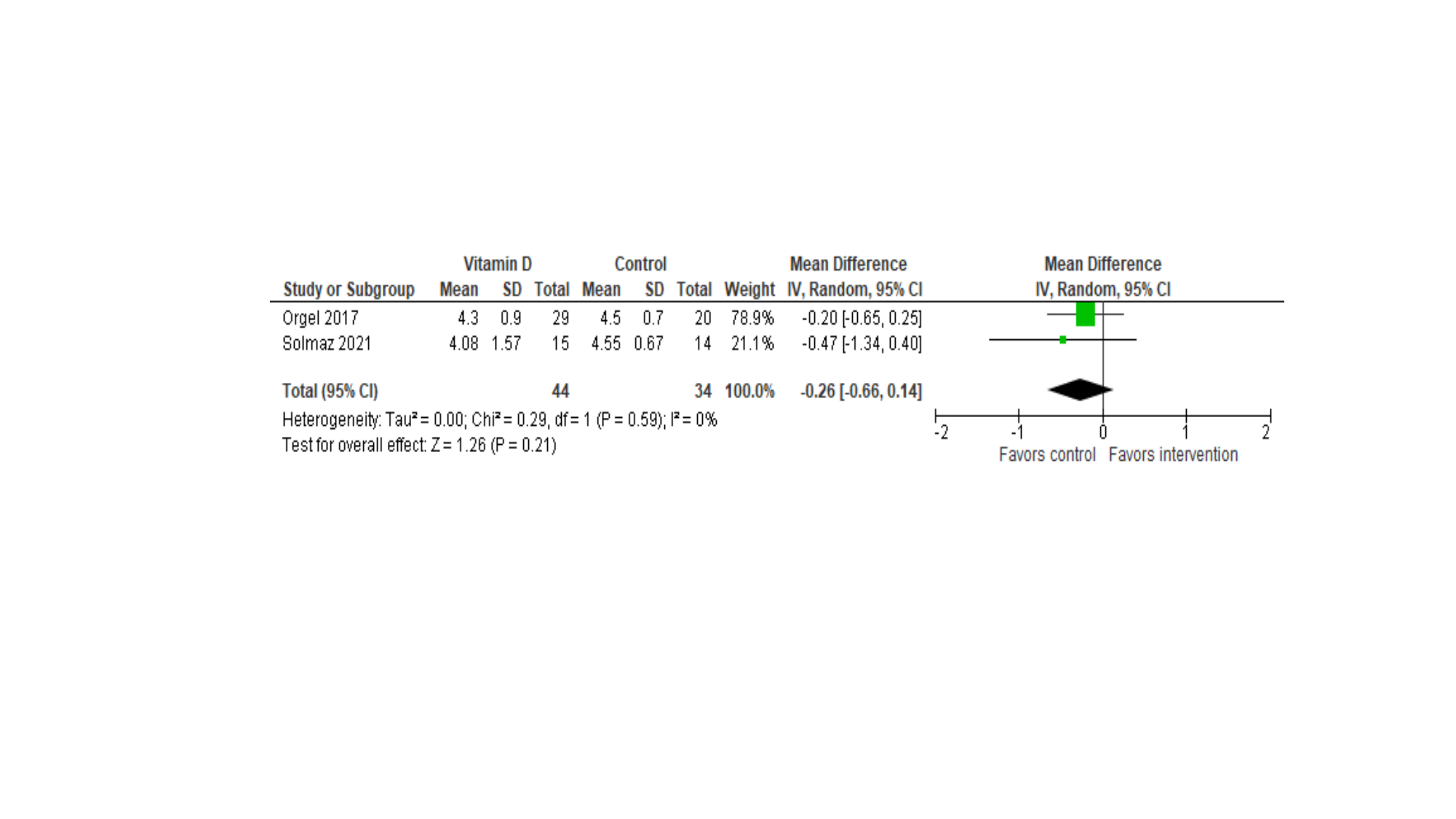

Supplement: Supplementary file 5 — Additional file 5. Figure S1. Risk of bias summary. Judgements about each risk of bias item for each included study. In the x axis are the 25 studies that met the inclusion criteria, and the y axis shows the different types of bias. In green are classified the studies that meet the criteria for a low risk of bias, in yellow those with an unclear risk of bias, and in red the ones with high risk of bias. Figure S2. Forest plot showing the effect of glutamine vs placebo on hospitalization days. Figure S3. Forest plot showing the effect of vitamin D supplementation vs placebo on serum calcium (Ca). Figure S4. Forest plot showing the effect of vitamin D supplementation vs placebo on serum phosphorus (P). [file 40795_2024_892_MOESM5_ESM.pptx]
